# TAS2R5 screening reveals biased agonism that fails to evoke internalization and downregulation resulting in attenuated desensitization

**DOI:** 10.1371/journal.pone.0315820

**Published:** 2025-02-13

**Authors:** Donghwa Kim, Hannah R. Strzelinski, Stephen B. Liggett

**Affiliations:** 1 Department of Medicine and the Center for Personalized Medicine, University of South Florida Morsani College of Medicine, Tampa, Florida, United States of America; 2 Department of Molecular Pharmacology and Physiology, University of South Florida Morsani College of Medicine, Tampa, Florida, United States of America; Indian Institute of Technology Kanpur, INDIA

## Abstract

The bitter taste receptor type 5 (TAS2R5) is expressed on multiple cell types and appears to be a suitable target for novel agonist treatments across multiple therapeutic areas. Like most G protein coupled receptors (GPCRs), TAS2R5 undergoes functional desensitization with prolonged agonist exposure which could limit effectiveness. The net loss of cellular receptors (termed downregulation) is a prominent mechanism of long-term desensitization; we screened 13 agonists for downregulation of receptor protein in TAS2R5-transfected HEK-293T and airway smooth muscle cells in culture, searching for pathway selectivity favoring G protein coupling over downregulation. The benchmark agonist 1,10-phenanthroline (denoted T5-1) evoked as much as 75% downregulation of TAS2R5 protein expression with 18-24 hrs of agonist exposure, while an analogue of T5-1 (denoted T5-12) caused a 2-3 fold *increase* in expression. Functionally, T5-1 and T5-12 were found to be full agonists when measuring [Ca^2+^]_i_ or ERK1/2 stimulation. The T5-12 phenotype was found to be due to agonist-induced stabilization of the receptor confining it to the cell membrane with subsequent failure to undergo internalization and receptor degradation. This occurred despite normal (referenced to T5-1) GRK-mediated receptor phosphorylation and β-arrestin recruitment by T5-12. Consistent with the lack of downregulation, T5-12 evoked much *less* functional desensitization of the [Ca^2+^]_i_ (43% vs 78%) and ERK1/2 (64% vs > 95%) responses compared to T5-1, respectively. We conclude that TAS2R5 pathway signaling is malleable to a more favorable therapeutic profile by agonist-receptor interactions that preserve primary signaling and minimizes desensitization.

## Introduction

G protein coupled receptors (GPCRs) are expressed on multiple cell types throughout the body, and indeed represent the largest superfamily of proteins. Signaling is initiated by agonist binding, leading to association with G protein and its dissociation into α and βγ subunits. The bound Gα subunit or the free βγ activate signaling by interaction with effectors such as phospholipase C (PLC) and adenylyl cyclase [1]. In some cases, the structure of the agonist can stabilize receptor conformations that differ from endogenous or “benchmark” agonists, leading to pathway selectivity, also referred to as agonist biasing [1,2]. Most GPCRs undergo desensitization (loss of function) upon chronic agonist exposure. Clinically this is exhibited as tachyphylaxis, and can limit the effectiveness of potential therapeutics targeting a given receptor [3]. The major mechanism of long-term (hrs) agonist-mediated desensitization is a net loss of cellular receptor protein expression, which is termed downregulation. A major component of downregulation for many GPCRs is initiated by receptor internalization which is the endocytosis of cell surface receptors to the cell interior, an event that leads to intracellular receptor protein degradation [3]. Early responses to agonist that promote short-term (minutes) desensitization are phosphorylation of Ser/Thr in the intracellular portions of the receptor, which in turn recruits binding of arrestins to the receptor. Bound arrestins compete with receptor-G protein binding (termed uncoupling), partially quenching receptor signaling. Arrestins also act as scaffolds that promote interactions between receptors and other cellular proteins. In the case of desensitization, they act to cause receptor internalization to the cell interior, which leads to downregulation with more prolonged agonist exposure. Thus the short-term desensitization events for a prototypical GPCR lead to the long-term events [3]. An agonist that is biased away from one or more events comprising functional desensitization, while maintaining G protein coupling, could result in less tachyphylaxis and thus a more favorable clinical profile [4]. In the current work, we define a type of agonist bias where G protein coupling at baseline is preserved, but the agonist is biased away from downregulation of receptor number and desensitization from long-term agonist exposure.

Bitter taste receptors (TAS2Rs) are GPCRs that act as chemoreceptors on many cell types outside the oral cavity and are potential targets for new therapeutics [5,6]. The first extraoral TAS2R was found by our group on human airway smooth muscle (HASM) cells, where they act to relax the muscle, and oppose the bronchoconstriction in obstructive lung diseases such as asthma [7,8]. The canonical signaling pathway of TAS2Rs, as identified in endogenously expressing [7,9] and transfected model cells [10], results in an increase in intracellular calcium [Ca^2+^]_i_ which occurs via the release βγ from Gi1,2,3 (or gustducin) [11] activating PLC leading to inositol-3-phosphate (IP3) generation. IP3 binds to the IP3 receptor on the endoplasmic reticulum releasing calcium stores. The increased [Ca^2+^]_i_ evokes specific responses depending on the cell type. Of the 25 TAS2R subtypes, TAS2R5 is highly expressed in HASM cells, and has been the target of drug discovery efforts [7,12,13]. With the aim of achieving structural diversity and improving potency, we have synthesized multiple novel compounds that activate TAS2R5 with a spectrum of potencies and efficacies [12]. In the current study we took advantage of this library to ascertain the potential for differential agonist-promoted receptor downregulation. We identified one agonist that not only failed to evoke downregulation, but actually increased TAS2R5 expression. This was found to be due to the agonist promoting conformation of the receptor that failed to internalize and subsequently downregulate.

## Materials and methods

### Cell culture and transfections

HEK-293T and D9 HASM [14] cells were cultured in Dulbecco’s modified Eagles’ medium with 10% fetal calf serum in a 37 °C, 95% air, 5% CO_2_ atmosphere. A previously described TAS2R expression construct [15] was utilized for transfections at 10-30 µg/10 cm^2^ dish with a ratio of 1.25 µL Lipofectamine 2000 (ThermoFisher, #11668027) per µg DNA. The next day cells were split into other culture dishes for specific assays typically performed 48 hrs after transfection at ~90% confluency.

### Test TAS2R5 agonists

The compounds utilized in this study and their sources have been previously described [12]. Unless otherwise noted, the concentrations of the compounds used in the various assays was 10-fold greater than the EC_50_ values obtained in dose-response studies of [Ca^2+^]_i_ stimulation [12]. This dose was used to avoid cell toxicity with the long-term incubations. For the two main compounds in the current study, these concentrations were 290 μM T5-1, and 120 μM T5-12. See S1 Fig for the structures and EC_50_ values for all compounds in the screen.

### Long-term desensitization of the [Ca^2+^] _i_ and p-ERK1/2 responses

The [Ca^2+^]_i_ response to GPCRs is transient, evoking multiple downstream events depending upon cell type. We utilized a standard pretreatment/wash/re-exposure protocol to ascertain the effects of 18 hrs of agonist exposure on TAS2R5 functional stimulation of [Ca^2+^]_i_. In all cases the [Ca^2+^]_i_ response to bradykinin, acting at its receptor, was also determined after TAS2R5 agonist exposure to confirm TAS2R5-specific (homologous) desensitization. Cells in 96 well plates were treated in the above media with vehicle or the indicated TAS2R5 agonist at 10X the EC_50_ for 18 hrs at 37 °C in the incubator, washed twice with calcium-free HBSS (Gibco, #14-175-095), and then loaded with Fluo-4 Direct Calcium Dye (Invitrogen, #F10471) as per the manufacturer’s protocol [16]. The dye was then aspirated and replaced with calcium-free HBSS supplemented with 2.5 mM probenecid. A Flexstation 3 (Molecular Devices) was used to acquire the [Ca^2+^]_i_ signal with an excitation wavelength of 485 nm and an acquisition wavelength of 515 nm. After establishing a baseline for 20 sec, drugs were automatically pipetted into the wells and measurements taken for the next 100 sec. To establish the response in the nondesensitized state, cells were pretreated with vehicle alone. For the TAS2R5 agonists the re-exposure dose was 30X the EC_50_; cells were also challenged with bradykinin (2 µM) and ionomycin (2 µM) as controls. Data was normalized as the % of the ionomycin response. The peak Ca^2 +^ response was determined by Prism software (GraphPad). Real-time ERK1/2 activation was determined in cultured HASM cells after overnight serum starvation followed by treatment with agonists at the indicated times [17]. Total proteins were separated by 12% sodium dodecyl sulfate–gel electrophoresis (SDS-PAGE) followed by immunoblots using antibodies for total ERK1/2 and phosphorylate ERK/1/2 (p-ERK1/2). The ratio of p-ERK1/2 to total ERK1/2 was taken as the measure of activation.

### Western blots

SDS-PAGE was performed over 6 hrs using 12% polyacrylamide gels on samples solubilized in a modified RIPA buffer (50 mM Tris-Hcl pH7.4, 100 mM NaCl, 1 mM EDTA, 1 mM EGTA, 1% Nonidet P-40, 1% TritonX-100, 0.25% sodium deoxycholate). The electrophoresed proteins were then transferred to PVDF membranes overnight. The primary antibodies for the western blots were: FLAG (Sigma-Aldrich, #F7425) at a titer of 1:1000, beta-actin (Sigma-Aldrich, #A1978) at a titer of 1:2000, ERK1/2 (Cell Signaling Technology, #9101 and #9102 for p-ERK1/2 and total ERK1/2, respectively) at titers of 1:1000. The secondary antibodies for chemiluminescence were #NA931V and #NA934V (GE Healthcare) at titers of 1:10000. After blocking, membranes were treated with primary antibody for 1.5 hrs, washed, and the treated with secondary antibody for 1.0 hr. ChemiDoc (Bio-Rad) was used to capture images which were quantitated using ImageJ (National Institutes of Health).

### Receptor phosphorylation and β-arrestin2 recruitment

For receptor phosphorylation, transfected HEK-293T were exposed to agonist for 10 min and lysed with modified RIPA buffer including protease and phosphatase inhibitor cocktails (GenDEPOT). The clarified total cell lysate was incubated with Phos-tag agarose beads (FUJIFILM Wako Chemical, #AG-503), at 4 °C overnight with constant rotation. Beads were washed four times with RIPA, and proteins released by adding 2X Laemmli sample buffer (Sigma-Aldrich, #S3401). Equivalent amounts of eluted proteins were subjected to SDS-PAGE, and immunoblots with the FLAG antibody were performed on the lysate (the input) or the precipitated proteins. For β-arrestin recruitment, β-Arr2-Rluc and GFP-CAAX expression constructs, extensively described by Bouvier and collegues [18], were transfected into TAS2R5 stably expressing HEK-293T cells using 8 µg of each construct. Here, β-Arr2-Rluc recruitment by TAS2R5 is detected by agonist-promoted increase in the proximity of the β-Arr2-Rluc (energy donor) moiety to GFP (energy acceptor) fused via its C-terminus to the CAAX box of KRas, which resides in the cell membrane where the receptor is embedded. Cells were cultured in 96-well plates with substrate (Prolume purple, NanoLight Tech, #369) and exposed to T5-1, T5-12, or vehicle for 10 min. BRET data was obtained as described [19] with signal aquisition at 420 nm and 515 nm using a FlexStation 3 (Molecular Devices).

### RT-PCR

RT-PCR was performed as described [20] using a LightCycler 96 system (Roche, Basel, Switzerland). The RT reactions utilized Oligo d(T)16 using M-MLV reverse transcriptase (M1708, Promega, Madison, WI, USA) and TaqMan Gene Expression master mix (2X) (Applied Biosystems, Foster City, CA, USA). The TaqMan primers were also from Applied Biosystems (TAS2R5: #Hs00251818_s1, GAPDH: #Hs03929097_g1). Quantification of transcripts was made by the deltaCt method.

### TAS2R5 internalization assay

FLAG-tagged TAS2R5 expressed in HEK-293T cells were treated with agonist for 18 hrs in a 10 cm dish under cell culture conditions. The attached cells were subsequently washed with cold PBS and then 10 mL of biotin solution consisting of 12 mg of sulfo-NHS-SS-Biotin (#21331, Thermo Fisher Scientific, Waltham, MA, USA) in 50 mL of cold PBS was added to the dish, which was then rocked for 60 min at 4 °C. The reaction was stopped by the addition of 500 µL of quenching solution (50 mM Glycine, 50 mM ammonium chloride). Cells were detached, washed twice by centrifugation, and then solubilized in 1 mL of modified RIPA buffer. 5% of the total lysate was saved for immunoblotting with FLAG which represented the input for immunoprecipitation. The remaining lysate was incubated with 30 µL of Avidin-agarose beads (#20219, Thermo Fisher Scientific, Waltham, MA, USA) overnight at 4 °C with constant rotation. The beads were washed three times with lysis buffer and the proteins were released by adding 2x Laemmli sample buffer. The entire immunoprecipitate was loaded for SDS-PAGE and immunoblotting with FLAG antibody. The ratio of the immunoprecipitated TAS2R5 to the input TAS2R5 was taken as the measure of the percent cell-surface receptors.

### Confocal microscopy

HEK-293T cells stably transfected to express the FLAG-tagged TAS2R5 were split onto coverslips at 35% confluency. The following day coverslips were treated with T5-1 (290 μM), T5-12 (120 μM), or vehicle for 10 hrs unders cell culture conditions (see above). After washing the coverslips with PBS, the attached cells were were fixed in 4% paraformaldehyde for 20 min, washed with PBS, and then permeabilized with 0.5% NP-40 for 20 min, and then washed again with PBS. Coverslips were blocked in 10% normal goat serum (Gibco, #PCN5000) for 45 min, then incubated with the primary antibodies (LAMP1, titer 1:100, Fisher #L1418; FLAG, titer 1:75, Fisher #B3111) for 2 hrs. Cells were then washed with PBS and incubated with fluorescent secondary antibodies (Fisher #A32766, titer 1:400 for the 488 nm emmission; Fisher #A32740, titer 1:400 for the 594 nm emission) for 45 minutes, and washed with PBS and then TBS. Nuclear staining was with DAPI (Fisher, #50874100001, 1 µg/mL) for 1 min. Coverslips were mounted on slides using ProLong Glass Antifade Mountant (Fisher, #P36982). Images were obtained using a Leica SP8 Confocal Microscope at a magnification of 90x and analyzed using ImageJ software (National Institutes of Health). Image channels were separated into red, green, and blue representing LAMP1, FLAG-TAS2R5, and DAPI, respectively. For colocalization, the LAMP1 and FLAG-TAS2R5 channels were merged, then a white colocalization signal was selected using the color thresholding tool set to hue 37–47. Six experiments were performed with each of the 3 conditions, with 4 fields per coverslip imaged and analyzed. Results are shown as arbitrary units which are derived from the acquired area of white images normalized to the number of cells (the blue area).

### Statistical analysis

Data are shown as mean ±  SE, or as representative results, as indicated. Statistical comparisons were performed using PRISM. Comparisons between groups were performed by ANOVA with post-hoc t-tests. Comparisons to a baseline were by Wilcoxon signed rank tests. Significance was imparted when P values were less than 0.05 from the above tests.

## Results and discussion

### A TAS2R5 agonist that fails to evoke agonist-promoted receptor downregulation

**Fig 1:**
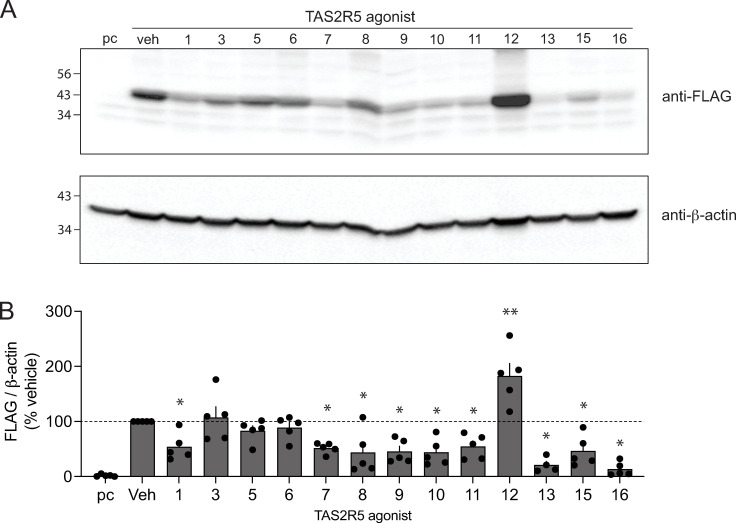
Screening TAS2R5 agonists for the receptor downregulation response to long- term agonist exposure. HEK-293T cells stably expressing FLAG-tagged TAS2R5 were exposed in culture for 18 hrs to the indicated agonists at a concentration 10-fold greater than their functional EC_50_ values. Western blots with FLAG antibody identified the receptor, with β-actin serving as the loading control. Nine of the 13 agonists showed a decrease in receptor expression, 3 showed no change, and 1 showed an increase in expression. A) a representative western blot; B) results from 4-5 experiments. See S1 Fig for structures and EC_50_ values. * , P < 0.05; **, P < 0.01 vs vehicle control. pc, plasmid control (empty vector).

**Fig 2:**
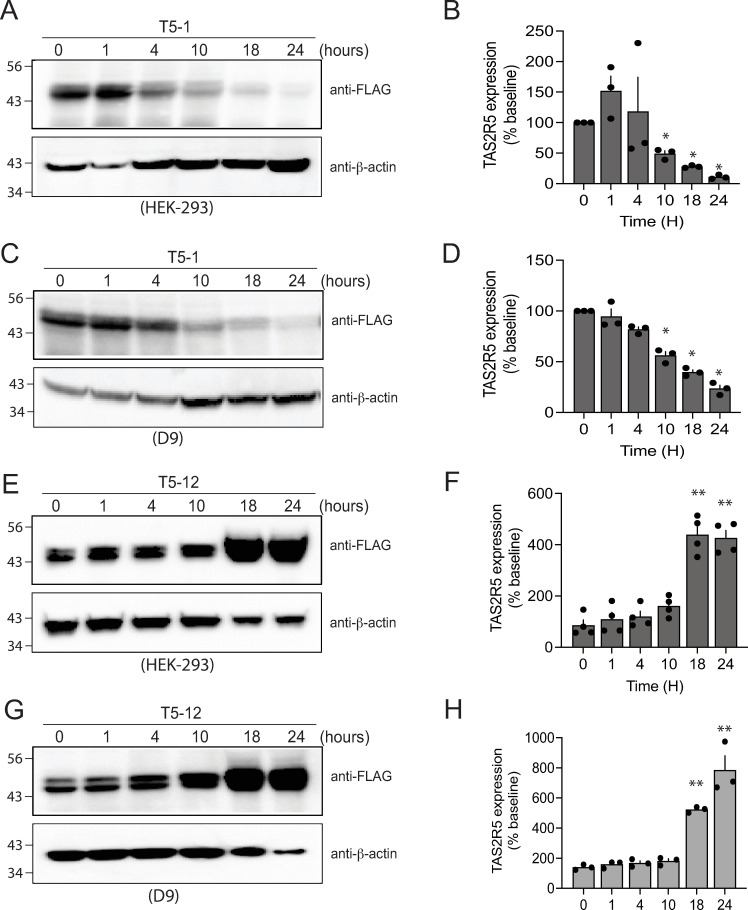
Regulation of receptor expression by T5-1 and T5-12 in two cell types. The time course of TAS2R5 regulation by T5-1 (A–D) and T5-12 (E–H) in stably transfected HEK-293T cells and D9 immortalized human airway smooth muscle cells. At the later time points T5-1 decreased TAS2R5 expression while T5-12 increased expression in both cell types. A, C, E, G) representative western blots, B, D, F, H) results from 3 experiments. * , P < 0.05; **, P < 0.01, vs baseline (t = 0).

HEK-293T cells stably transfected to express FLAG-TAS2R5 were treated in culture for 18 hrs with each of 13 agonists (S1 Fig, denoted T5-1, T5-2, etc) at a concentration that is 10-fold greater than their EC_50_ values [12] for stimulating [Ca^2+^]_i_. Cell membranes were subjected to SDS-PAGE and immunoblotting with FLAG antibody. As shown in Fig 1, one agonist (T5-12) evoked an upregulation of TAS2R5 protein, which might be a phenotype that abrogates long-term functional desensitization of the cellular response. Subsequent studies were performed comparing the T5-12 agonist with the benchmark [10] TAS2R5 agonist T5-1, which in the screening studies displayed ~40% downregulation. Time-based experiments (Fig 2) of agonist exposures of 1–24 hrs were performed with the transfected HEK-293T cells as well as transfected D9 HASM cells, which is a cell type of interest for potential therapeutics in airway disease. T5-1 invoked downregulation of TAS2R5 protein that was first observed at 4–10 hrs in HEK-293T cells with a continued decrease in expression over time amounting to ~ 90% loss of expression by 24 hrs (Fig 2A and B). A similar pattern for T5-1 was observed in the D9 HASM cells (Fig 2C and D). In contrast, T5-12 exhibited upregulation at the later time points, in both cell types, amounting to at least 3-fold over baseline (Fig 2E–H).

### The T5-12 phenotype is related to post-translational processes

**Fig 3:**
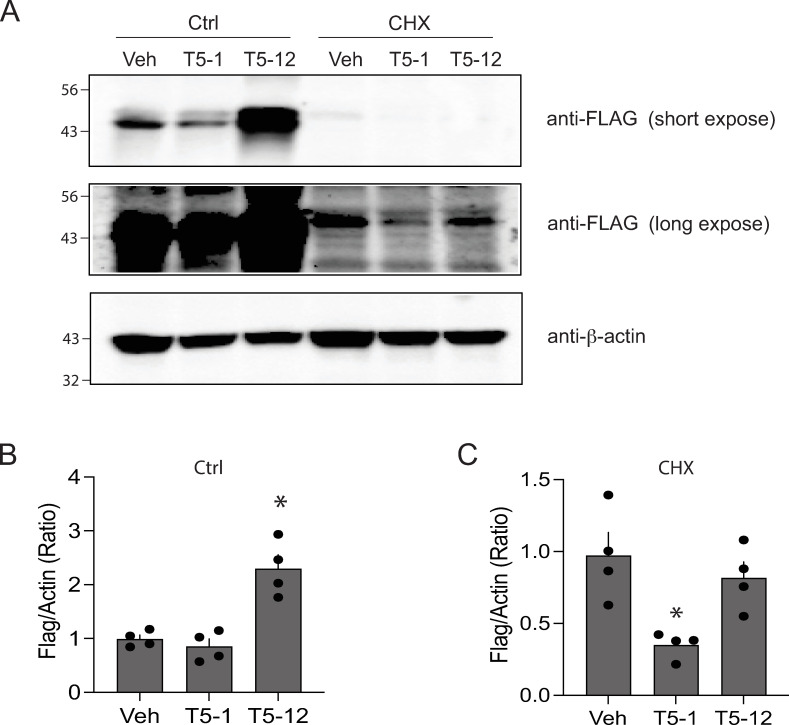
Effects of T5-1 and T5-12 on TAS2R5 expression during protein synthesis inhibition. A) A representative immunoblot. Cells were treated with agonist for 18 hrs. The receptor expression responses in normal media are found in the left 3 lanes, and in the presence of cycloheximide (CHX) in the right 3 lanes. To clearly show the bands, two exposures are shown with the FLAG antibody (short =  35 sec, long =  500 sec). B and C) Results from 4 experiments. In the presence of CHX, T5-1 caused a decrease in receptor expression with the prolonged exposure, while T5-12 exposure had no significant effect on expression. * , P < 0.01 vs vehicle control.

To assess whether this upregulation by T5-12 is due to elevated TAS2R5 mRNA, quantitative RT-PCR was performed with the D9 HASM cells treated for 18 hrs with T5-1 or T5-12. There was no significant change in TAS2R5 transcript levels with the T5-1 treatment, while T5-12-treated cells actually showed a decrease in TAS2R5 mRNA (S2 Fig). Since the upregulation of TAS2R5 protein expression by T5-12 is accompanied by a decrease in TAS2R5 transcripts, we conclude that this agonist’s novel effect is not at the level of transcription or mRNA stability. We next assessed the effects of blocking protein synthesis on the T5-12 effect on TAS2R protein expression. Cells were treated with the protein synthesis inhibitor cycloheximide (CHX), and as expected, there was a decrease in TAS2R5 protein in the absence of agonist, indicating an ongoing degradation process (Fig 3A, see vehicle control vs vehicle CHX). We then determined whether T5-12 blunted this degradation, suggesting that this agonist-receptor pair is “locked” and does not proceed to internalization or subsequent downregulation. Cells were treated for 18 hrs with vehicle, T5-1, or T5-12 with or without CHX. Under the CHX conditions, T5-1 decreased TAS2R5 protein expression; in contrast, T5-12 treatment resulted in no decrease in expression (Fig 3B). These results indicate 1) that differential receptor protein *synthesis* is not the basis for the T5-1 vs T5-12 effects on TAS2R5 expression and 2) suggest that the T5-12 bound TAS2R5 represents a unique conformation of the complex, which is less amenable to downregulation.

### T5-12 fails to promote TAS2R5 internalization and co-localization with lysosomes

**Fig 4:**
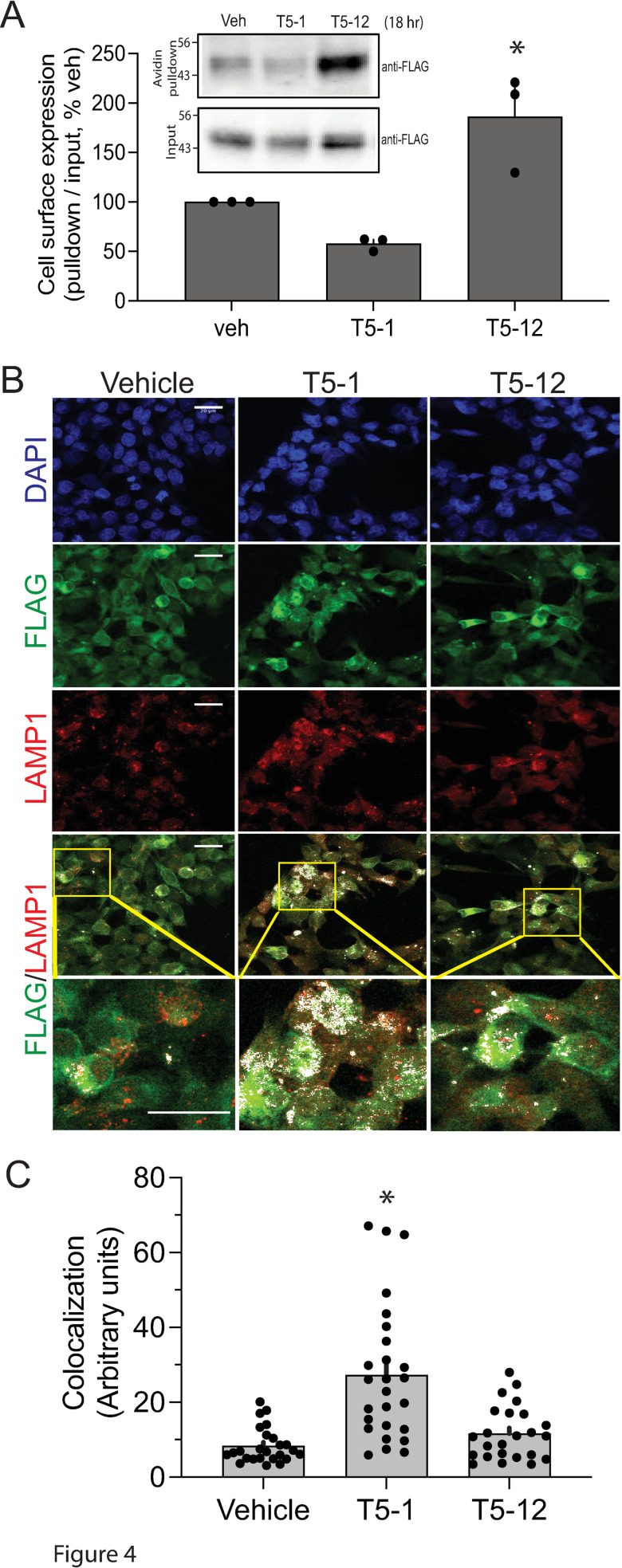
T5-12 fails to promote TAS2R5 internalization and colocalization with lysosomes. A) Cell surface expression of FLAG-tagged TAS2R5 expressed in HEK-293T cells was determined using a biotinylation assay (see Methods). Cell surface proteins were covalently labeled with biotin, treated with agonists T5-1 and T5-12 for 18 hrs, lysed, immunoprecipitated with avidin, and the pulldowns probed by immunoblots blots using the FLAG antibody. A representative blot is shown in the inset, and results from 4 experiments are depicted in the bar graph. * , P < 0.05 vs vehicle treatment. B) Representative confocal images of stably transfected cells treated with T5-1 or T5-12 for 18 hrs. DAPI staining (blue) represents the nucleus, TAS2R5 emits a green signal, and LAMP1 emits a red signal. Colocalization of TAS2R5 with LAMP1 is represented by the merged signals which are white. The bottom row of colocalization images is shown with increased magnification to highlight the differences between the 2 agonists. Bar =  20 µm. C) Quantitative results from 6 confocal experiments, each visualizing 4 random fields (~50 cells per field) per coverslip at 90x magnification. * , P < 0.01 vs vehicle.

Since agonist-promoted receptor internalization precedes the protein degradation/downregulation events, we quantitated the loss of cell-surface TAS2R5 by the two agonists after 18 hrs of exposure using a biotinylation assay, which identifies cell surface FLAG-tagged TAS2Rs. T5-1 treated cells underwent a ~ 40% loss of cell-surface expression (quantitated as the ratio of cell-surface to total receptor expression) while T5-12 treatment showed no loss, and in fact a gain in cell surface expression (Fig 4A). The data also confirm that the accumulation of TAS2R5 receptors results in enhanced protein expression at the cell surface; we would thus expect the phenotype to have functional significance, such as attenuation of agonist-promoted desensitization. Given that TAS2R5 receptor protein failed to decrease with prolonged exposure to the T5-12 agonist or to engage the entry path (internalization) for agonist-promoted degradation, we considered that TAS2R5 would not colocalize with the lysosomal marker LAMP1 when cells were treated with T5-12. And, that such colocalization with this marker would be evident when cells were treated with T5-1 which does promote internalization and downregulation. Confocal microscopy (Fig 4B and C) indeed revealed a significant difference in TAS2R5-LAMP1 colocalization (white signals) in the cell interior between the two drug treatments. As shown, there is some co-localization in the absence of agonist, which is not unexpected since there is some degree of receptor turnover at baseline. However, cells treated for 18 hrs with T5-12 had no significant change in the extent of co-localization, which is in contrast to the increase observed with T5-1 treatment. These results confirm the different fates of TAS2R5 that are directed by agonist activation between T5-1 and T5-12, and are consistent with the lack of TAS2R5 downregulation by T5-12.

### T5-12 evokes less desensitization than T5-1 consistent with altered downregulation

**Fig 5:**
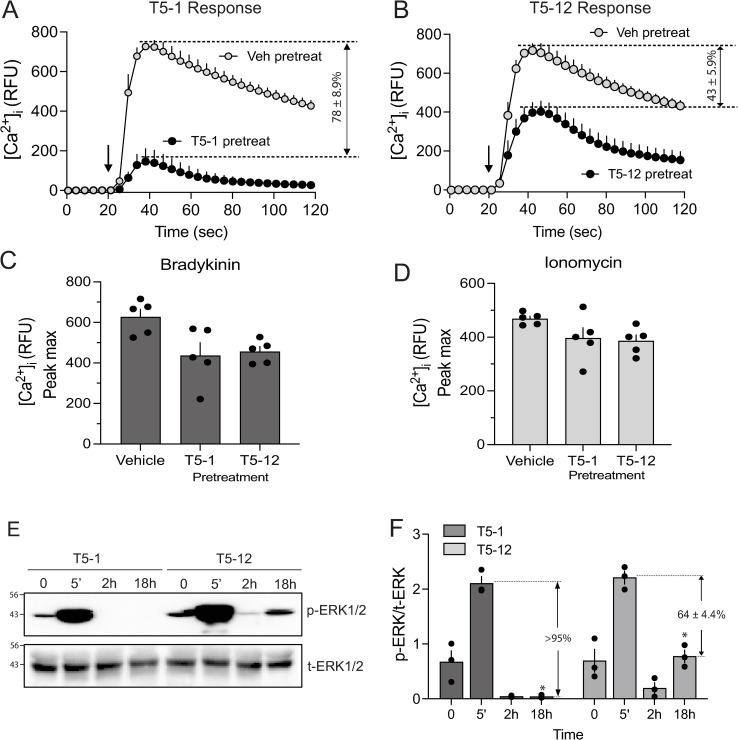
Agonist-promoted functional desensitization of TAS2R5 by T5-12 is blunted compared to T5-1. A) D9 HASM cells were pretreated with vehicle, T5-1 or T5-12 for 18 hrs, washed, and acutely exposed to the same agonists with [Ca^2+^]_i_ measured over the next 100 sec. The peak [Ca^2+^]_i_ stimulation by the two agonists was the same in vehicle treated cells. T5-1 stimulated peak [Ca^2+^]_i_ was markedly reduced by T5-1 pretreatment, representing ~ 78% desensitization. In contrast, T5-12 pretreatment caused a ~ 43% loss of function (P < 0.01 T5-12 vs T5-1). The [Ca^2+^]_i_ response to bradykinin (C) and ionomycin (D) were not differentially affected by the two agonists. E and F) Differential desensitization of ERK1/2 activation by T5-1 and T5-12. D9 HASM cells were treated for the indicated times with the agonists and the solubilized lysates probed for p-ERK1/2 or total ERK1/2 by western blots. The response at 5 min was equivalent between the two agonists. At later time points p-ERK1/2 was nearly undetectable in T5-1 treated cells, indicative of substantial (>95%) desensitization. T5-12 p-ERK1/2 levels were readily apparent at the 18 hrs time point representing ~ 64% desensitization E), representative western blot; F), results from 4 experiments, *  (P < 0.001 T5-1vs T5-12 levels at 18 hrs).

To address the signaling consequences of this lack of downregulation of TAS2R5 by T5-12, we measured the acute increase in [Ca^2+^]_i_ as a readout for receptor-coupled functional resposnes to the TAS2R5 agonists. Cells were pretreated with vehicle (control) or with the two agonists for 18 hrs. Cells were then washed and acutely exposed to the same agonist with immediate acqusition of the [Ca^2+^]_i_ signal over the next 100 sec (see Methods). The expectation was that this lack of the downregulation mechanism would partially impair desensitization. To assess any heterologous (non-receptor) desensitization, vehicle and TAS2R5 agonist-treated cells were also acutely stimulated with bradykinin which acts through its receptor to stimulate [Ca^2+^]_i_ in a G protein dependent manner, or the non-specific ionophore ionomycin. As indicated in Fig 5A and B, T5-1 pretreatment caused a ~ 78% loss of T5-1 stimulated signaling to [Ca^2+^]_i_. In contrast, T5-12 evoked ~ 43% desensitization (P < 0.001), indicating that the lack of downregulation has signaling implications for long-term desensitization. Fig 5C and D shows that the stimulation of [Ca^2+^]_i_ by bradykinin or ionomycin were not differentially affected by the two TAS2R5 agonists.

TAS2R5 also signals to the activation (phosphorylation) of ERK1/2 [11]. Functional assays designed to examine desensitization of this signal in real time also showed an attenuated desensitization by T5-12 (Fig 5E and F). While both agonists activated ERK1/2 with 5 min of exposure, the signal was markedly decreased at later time points with T5-1 exposure. In contrast, signaling by T5-12 was still evident after 18 hrs of exposure, consistent with the lack of downregulation (or in some models an upregulation) of TAS2R5. The ERK1/2 activation by T5-12 at 18 hrs was, however, less than the acute response. This is consistent, though, with some desensitization processes being maintained with T5-12, as was also shown in the [Ca^2+^]_i_ experiments (Fig 5B).

**Fig 6:**
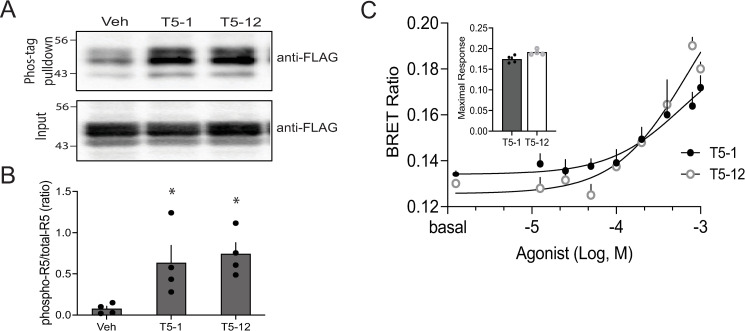
Agonist-promoted TAS2R5 phosphorylation and β-arrestin2 recruitment by T5-1 and T5-12 are equivalent. A) Representative result from a whole-cell TAS2R5 phosphorylation experiment. B) Mean results from 4 phosphorylation experiments. C) Representative dose-response experiment using BRET to determine β-arrestin2 recruitment by the two agonists. The inset shows the peak recruitment from 4 such experiments.

Differential T5-12 promoted phosphorylation of the receptor by GRKs or differential recruitment of β-arrestin might expain the lack of downregulation by this agonist, since these events preceed receptor degredation. Receptor phosphorylation by the two agonists was studied by exposing intact cells to the two agonists for 10 min then immunoprecipitated with a phosphotag antibody followed by immunoblotting with FLAG antibody. Both T5-1 and T5-12 induced an increase in receptor phosphorylation over vehicle, which was equivalent between the two agonists (Fig 6A and B). β-arrestin recruitment by agonist exposure for 10 min was measured using a BRET-based method [18]. Both T5-1 and T5-12 promoted β-arrestin2 recruitment to the same extent (Fig 6C). (Nontransfected cells showed no BRET signal, S3 Fig.) Taken together, these data indicate that these two events, which are precursers to downregulation, are not impaired with T5-12, and that aberrancies at these junctures do not account for the T5-12 phenotype. They also are consistent with the relatively small but consistent degree of agonist-promoted desensitization we observed with with T5-12 (in both the [Ca^2+^]_i_ and the ERK1/2 functional assays), since the events leading to receptor-G protein uncoupling (receptor phosphorylation and β-arrestin recruitment) are intact.

A developing theme for some GPCRs is the potential for ligand biasing to improve therapeutic efficacy. With receptors where biased agonists have been found, such as μOR [21], type 1 AngIIR [22], several muscarinic receptor subtypes [23] and β_2_AR [4], signaling to one pathway is maintained but the capacity for activation of a second pathway is blunted, compared to a benchmark balanced agonist [1,2,24,25]. In this context, the individual “signals” of interest are dependent upon the pathobiology of the disease being treated, where elimination of one pathway would be advantageous. Such signals can be highly discreet, or, involve several processes leading to a phenotype, such as agonist-promoted downregulation. The mechanism of the bias can occur at any point along the pathway. For a number of GPCRs, agonists have been described where G protein signaling is intact while signaling to β-arrestin is reduced [21], while other agonists have the opposite effect and recruit β-arrestin but have little G protein signaling [22]. Depending on the disease, either direction of β-arrestin biasing might be therapeutically desirable. This type of bias is only one of many that have been described (reviewed elsewhere) [1,2,23]. In the current study the bias is away from the internalization process, which leads to a lack of long-term agonist-promoted downregulation and thus substantially less desensitization. This phenotype is not due to a lack of GRK-mediated phosphorylation or β-arrestin recruitment, but appears to be related to subsequent steps in the process. We show that the receptor bound to T5-12 is not amenable to internalization compared to when T5-1 is bound. The T5-12 effect is manifested as a “locked” receptor, able to signal to Gi, and to β-arrestin, but does not appear to promote receptor endocytosis which would lead to receptor degradation. The lack of degradation with continued synthesis results in no appreciable loss of net receptor expression, and in fact an increase over time under some experimental conditions. Nevertheless, there is evidence of some degree of desensitization of the cellular response to T5-12 at these later time points, which is attributed to the β-arrestin uncoupling effect that remains intact. We note that the magnitude of the effect on functional desensitization observed with T5-12 is not trivial. In the Ca^2 +^ studies, both agonists achieve similar maximal responses in the absence of pretreatment (Fig 5A and B). After the long-term exposure, the magnitude of the Ca^2 +^ signal is ~ 2-fold greater with T5-12 compared to T5-1 (Fig 5A and B). For the ERK1/2 activation signal, T5-1-dependent signaling was essentially absent with 18 hrs of exposure, but with T5-12 ERK1/2 activation was observed at this time point, amounting to ~30% of the peak early response. In conclusion, the screening of multiple TAS2R5 ligands uncovered one agonist that fails to display key receptor regulatory events that are typically present with TAS2R5 and other GPCRs, leading to a response profile that would be advantageous since tachyphylaxis would be significantly blunted.

## Supporting information

S1 Fig Chemical structures of the test compounds. Shown are the structures of the 13 compounds that were screened in Fig 1. The EC_50_ values that are indicated were from [Ca^2+^]_i_ stimulation experiments [12].
(TIF)

S2 Fig Effects of T5-1 and T5-12 on TAS2R5 mRNA expression levels after exposure to the agonists for 18 hrs. Results are from 9 experiments *P < 0.01
(TIF)

S3 Fig Nontransfected HEK-293T cells show no agonist-promoted BRET signal. As a control, HEK-293T cells that were not transfected with TAS2R5 or the two biosensors (β-arrestin2-Rluc and GFP-CAAX) were otherwise subjected to the experimental protocol for BRET (see Methods). Neither T5-1 or T5-12 resulted in a change in the luciferase or GFP signals, consistent with no BRET signal. Results are from 3 experiments.
(TIF)

S1 Raw Images from multiple western blot assays. (PDF)

## References

[1] Kim D, Tokmakova A, Woo J-AA, An SS, Goddard WA 3rd, Liggett SB. Selective signal capture from multidimensional GPCR outputs with biased agonists: progress towards novel drug development. Mol Diagn Ther. 2022;26(4):383–96. doi: 10.1007/s40291-022-00592-4 35595932 PMC9276727

[2] Tokmakova A, Kim D, Goddard WA 3rd, Liggett SB. Biased β-agonists favoring Gs over β-arrestin for individualized treatment of obstructive lung disease. J Pers Med. 2022;12(3):331. doi: 10.3390/jpm12030331 35330331 PMC8955194

[3] Rajagopal S, Shenoy SK. GPCR desensitization: acute and prolonged phases. Cell Signal. 2018;41:9–16. doi: 10.1016/j.cellsig.2017.01.024 28137506 PMC5533627

[4] Kim D, Tokmakova A, Lujan LK, Strzelinski HR, Kim N, Najari Beidokhti M, et al. Identification and characterization of an atypical Gαs-biased β2AR agonist that fails to evoke airway smooth muscle cell tachyphylaxis. Proc Natl Acad Sci U S A. 2021;118(49):e2026668118. doi: 10.1073/pnas.2026668118 34857633 PMC8670521

[5] An SS, Liggett SB. Taste and smell GPCRs in the lung: evidence for a previously unrecognized widespread chemosensory system. Cell Signal. 2018;41:82–8. doi: 10.1016/j.cellsig.2017.02.002 28167233 PMC5939926

[6] Tuzim K, Korolczuk A. An update on extra-oral bitter taste receptors. J Transl Med. 2021;19(1):440. doi: 10.1186/s12967-021-03067-y 34674725 PMC8529754

[7] Deshpande DA, Wang WCH, McIlmoyle EL, Robinett KS, Schillinger RM, An SS, et al. Bitter taste receptors on airway smooth muscle bronchodilate by localized calcium signaling and reverse obstruction. Nat Med. 2010;16(11):1299–304. doi: 10.1038/nm.2237 20972434 PMC3066567

[8] Robinett KS, Koziol-White CJ, Akoluk A, An SS, Panettieri RA Jr, Liggett SB. Bitter taste receptor function in asthmatic and nonasthmatic human airway smooth muscle cells. Am J Respir Cell Mol Biol. 2014;50(4):678–83. doi: 10.1165/rcmb.2013-0439RC 24219573 PMC4068928

[9] Robinett KS, Deshpande DA, Malone MM, Liggett SB. Agonist-promoted homologous desensitization of human airway smooth muscle bitter taste receptors. Am J Respir Cell Mol Biol. 2011;45(5):1069–74. doi: 10.1165/rcmb.2011-0061OC 21642585 PMC3361362

[10] Meyerhof W. Elucidation of mammalian bitter taste. Rev Physiol Biochem Pharmacol. 2005;154 37–72. doi: 10.1007/s10254-005-0041-0 16032395

[11] Kim D, Woo JA, Geffken E, An SS, Liggett SB. Coupling of airway smooth muscle bitter taste receptors to intracellular signaling and relaxation is via Gαi1,2,3. Am J Respir Cell Mol Biol. 2017;56(6):762–71. doi: 10.1165/rcmb.2016-0373OC 28145731 PMC5516295

[12] Kim D, An SS, Lam H, Leahy JW, Liggett SB. Identification and characterization of novel bronchodilator agonists acting at human airway smooth muscle cell TAS2R5. ACS Pharmacol Transl Sci. 2020;3(6):1069–75. doi: 10.1021/acsptsci.0c00127 33344890 PMC7737212

[13] Yang MY, Kim S-K, Kim D, Liggett SB, Goddard WA 3rd. Structures and agonist binding sites of bitter taste receptor TAS2R5 complexed with Gi protein and validated against experiment. J Phys Chem Lett. 2021;12(38):9293–300. doi: 10.1021/acs.jpclett.1c02162 34542294 PMC8650975

[14] Gosens R, Stelmack GL, Dueck G, McNeill KD, Yamasaki A, Gerthoffer WT, et al. Role of caveolin-1 in p42/p44 MAP kinase activation and proliferation of human airway smooth muscle. Am J Physiol Lung Cell Mol Physiol. 2006;291(3):L523-34. doi: 10.1152/ajplung.00013.2006 16617096

[15] Kim D, Pauer SH, Yong HM, An SS, Liggett SB. β2-Adrenergic receptors chaperone trapped bitter taste receptor 14 to the cell surface as a heterodimer and exert unidirectional desensitization of taste receptor function. J Biol Chem. 2016;291(34):17616–28. doi: 10.1074/jbc.M116.722736 27342779 PMC5016158

[16] Tokmakova A, Kim D, Guthrie B, Kim S-K, Goddard WA 3rd, Liggett SB. Predicted structure and cell signaling of TAS2R14 reveal receptor hyper-flexibility for detecting diverse bitter tastes. iScience. 2023;26(4):106422. doi: 10.1016/j.isci.2023.106422 37096045 PMC10121769

[17] Kim D, Cho S, Castaño MA, Panettieri RA, Woo JA, Liggett SB. Biased TAS2R Bronchodilators Inhibit Airway Smooth Muscle Growth by Downregulating Phosphorylated Extracellular Signal-regulated Kinase 1/2. Am J Respir Cell Mol Biol. 2019;60(5):532–40. doi: 10.1165/rcmb.2018-0189OC 30365340 PMC6503617

[18] Namkung Y, Le Gouill C, Lukashova V, Kobayashi H, Hogue M, Khoury E, et al. Monitoring G protein-coupled receptor and β-arrestin trafficking in live cells using enhanced bystander BRET. Nat Commun. 2016;7 12178. doi: 10.1038/ncomms12178 27397672 PMC4942582

[19] McGraw DW, Mihlbachler KA, Schwarb MR, Rahman FF, Small KM, Almoosa KF, et al. Airway smooth muscle prostaglandin-EP1 receptors directly modulate beta2-adrenergic receptors within a unique heterodimeric complex. J Clin Invest. 2006;116(5):1400–9. doi: 10.1172/JCI25840 16670773 PMC1451203

[20] Small KM, Brown KM, Seman CA, Theiss CT, Liggett SB. Complex haplotypes derived from noncoding polymorphisms of the intronless alpha2A-adrenergic gene diversify receptor expression. Proc Natl Acad Sci U S A. 2006;103(14):5472–7. doi: 10.1073/pnas.0601345103 16567612 PMC1459379

[21] DeWire SM, Yamashita DS, Rominger DH, Liu G, Cowan CL, Graczyk TM, et al. A G protein-biased ligand at the μ-opioid receptor is potently analgesic with reduced gastrointestinal and respiratory dysfunction compared with morphine. J Pharmacol Exp Ther. 2013;344(3):708–17. doi: 10.1124/jpet.112.201616 23300227

[22] Violin JD, DeWire SM, Yamashita D, Rominger DH, Nguyen L, Schiller K, et al. Selectively engaging β-arrestins at the angiotensin II type 1 receptor reduces blood pressure and increases cardiac performance. J Pharmacol Exp Ther. 2010;335(3):572–9. doi: 10.1124/jpet.110.173005 20801892

[23] Kaoullas MG, Thal DM, Christopoulos A, Valant C. Ligand bias at the muscarinic acetylcholine receptor family: Opportunities and challenges. Neuropharmacology. 2024;258:110092. doi: 10.1016/j.neuropharm.2024.110092 39067666

[24] Violin JD, Crombie AL, Soergel DG, Lark MW. Biased ligands at G-protein-coupled receptors: promise and progress. Trends Pharmacol Sci. 2014;35(7):308–16. doi: 10.1016/j.tips.2014.04.007 24878326

[25] Tóth AD, Turu G, Hunyady L. Functional consequences of spatial, temporal and ligand bias of G protein-coupled receptors. Nat Rev Nephrol. 2024;20(11):722–41. doi: 10.1038/s41581-024-00869-3 39039165

