# Supplementary figures and images for: TAS2R5 screening reveals biased agonism that fails to evoke internalization and downregulation resulting in attenuated desensitization
Source: PLoS One. 2025 Feb 13;20(2):e0315820. doi: 10.1371/journal.pone.0315820 (PMC11824966; doi:10.1371/journal.pone.0315820)

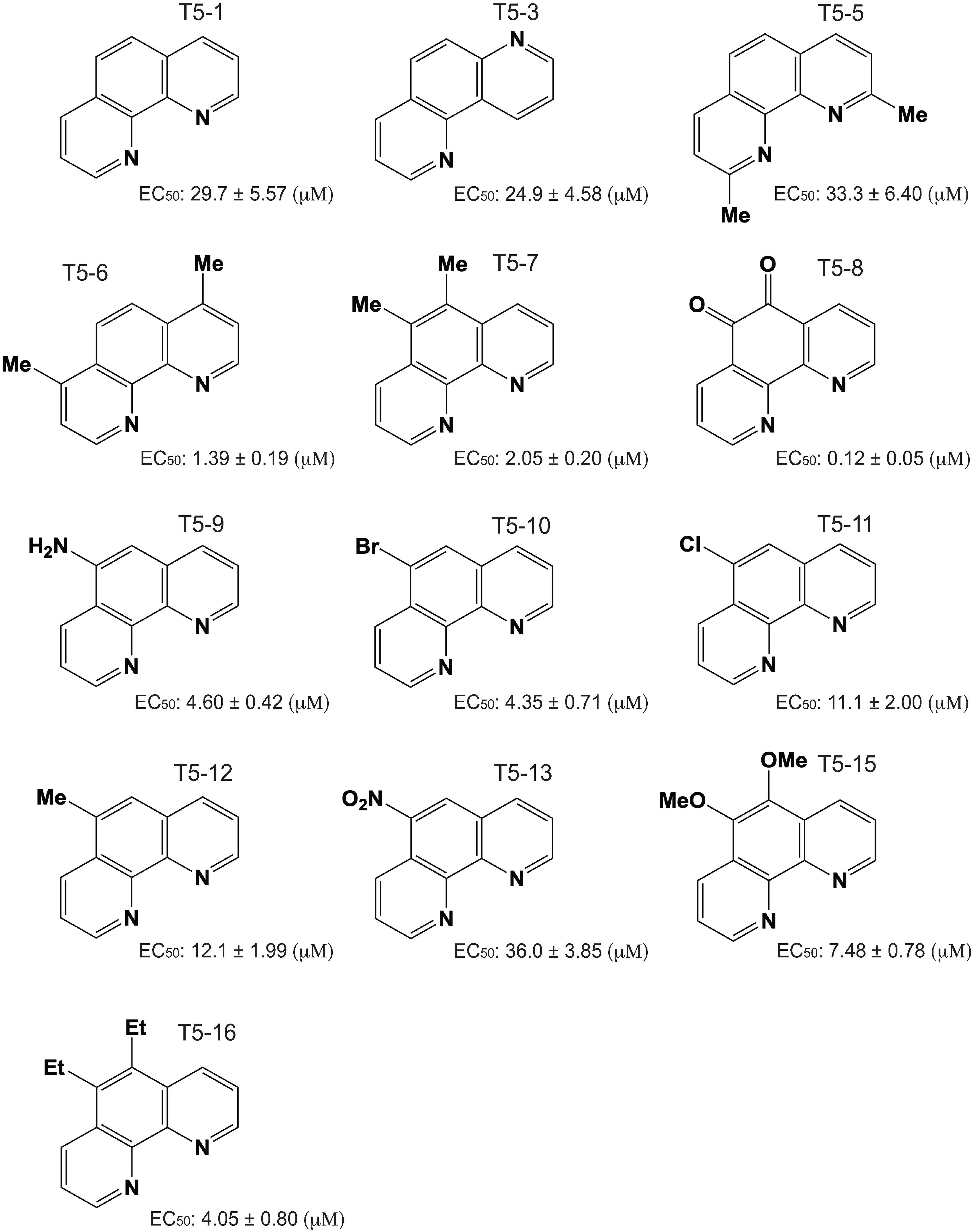

Supplement: S1 Fig — Shown are the structures of the 13 compounds that were screened in Fig 1. The EC50 values that are indicated were from [Ca2+]i stimulation experiments [12]. (TIF) [file pone.0315820.s001.tif]

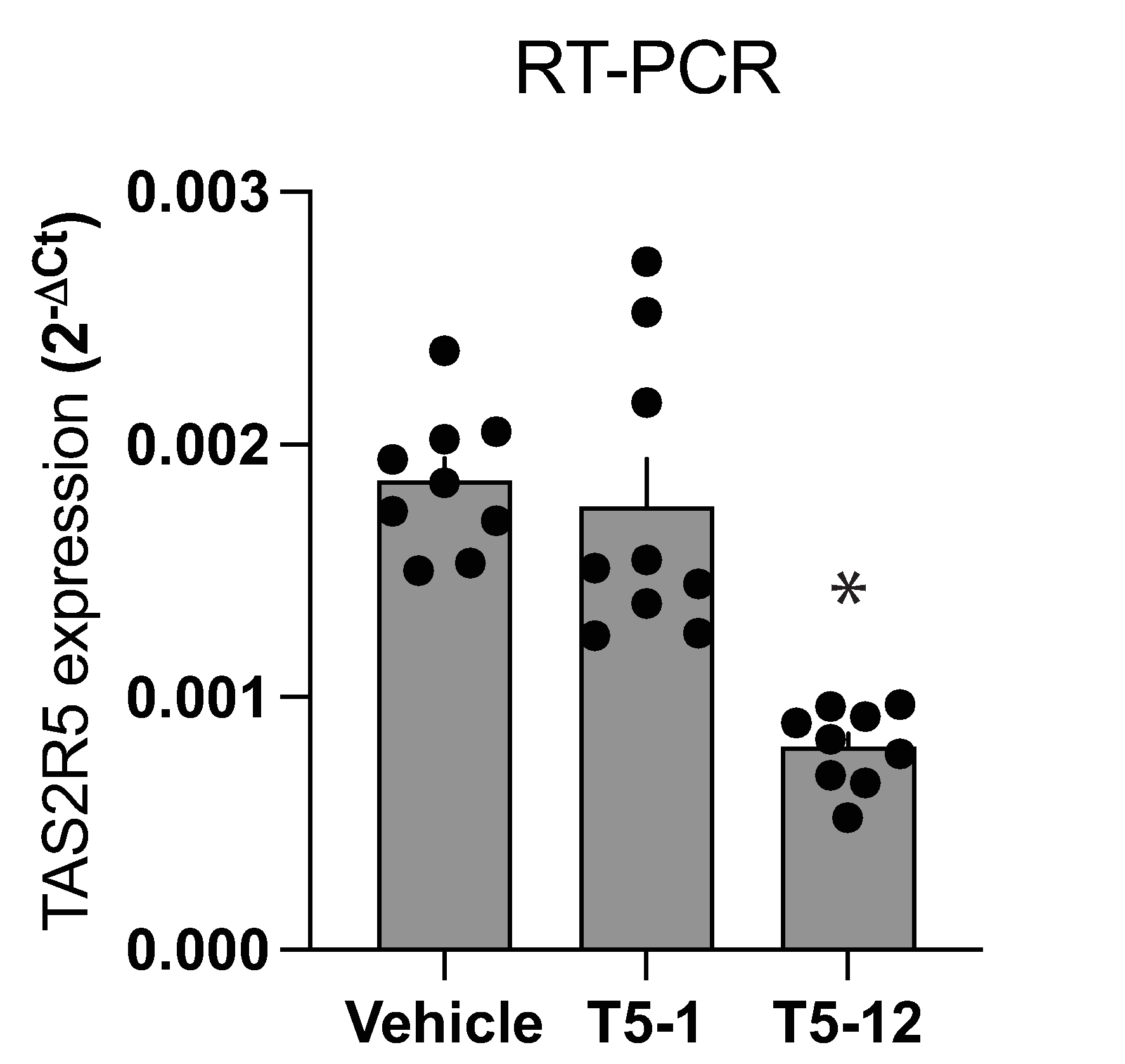

Supplement: S2 Fig — Results are from 9 experiments *P < 0.01 (TIF) [file pone.0315820.s002.tif]

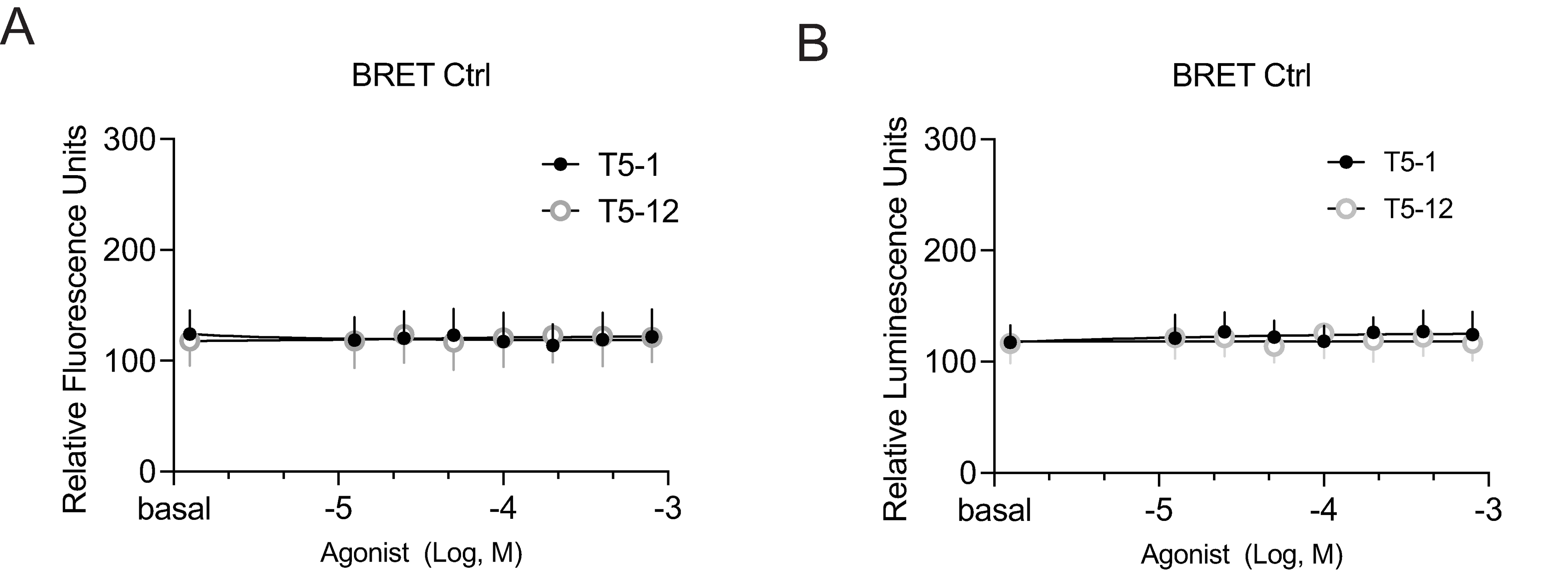

Supplement: S3 Fig — As a control, HEK-293T cells that were not transfected with TAS2R5 or the two biosensors (β-arrestin2-Rluc and GFP-CAAX) were otherwise subjected to the experimental protocol for BRET (see Methods). Neither T5-1 or T5-12 resulted in a change in the luciferase or GFP signals, consistent with no BRET signal. Results are from 3 experiments. (TIF) [file pone.0315820.s003.tif]
