# Supplementary material for: TAS2R5 screening reveals biased agonism that fails to evoke internalization and downregulation resulting in attenuated desensitization
Source: PLoS One. 2025 Feb 13;20(2):e0315820. doi: 10.1371/journal.pone.0315820 (PMC11824966; doi:10.1371/journal.pone.0315820)
Supplement: S1 Raw — (PDF) [file pone.0315820.s004.pdf]

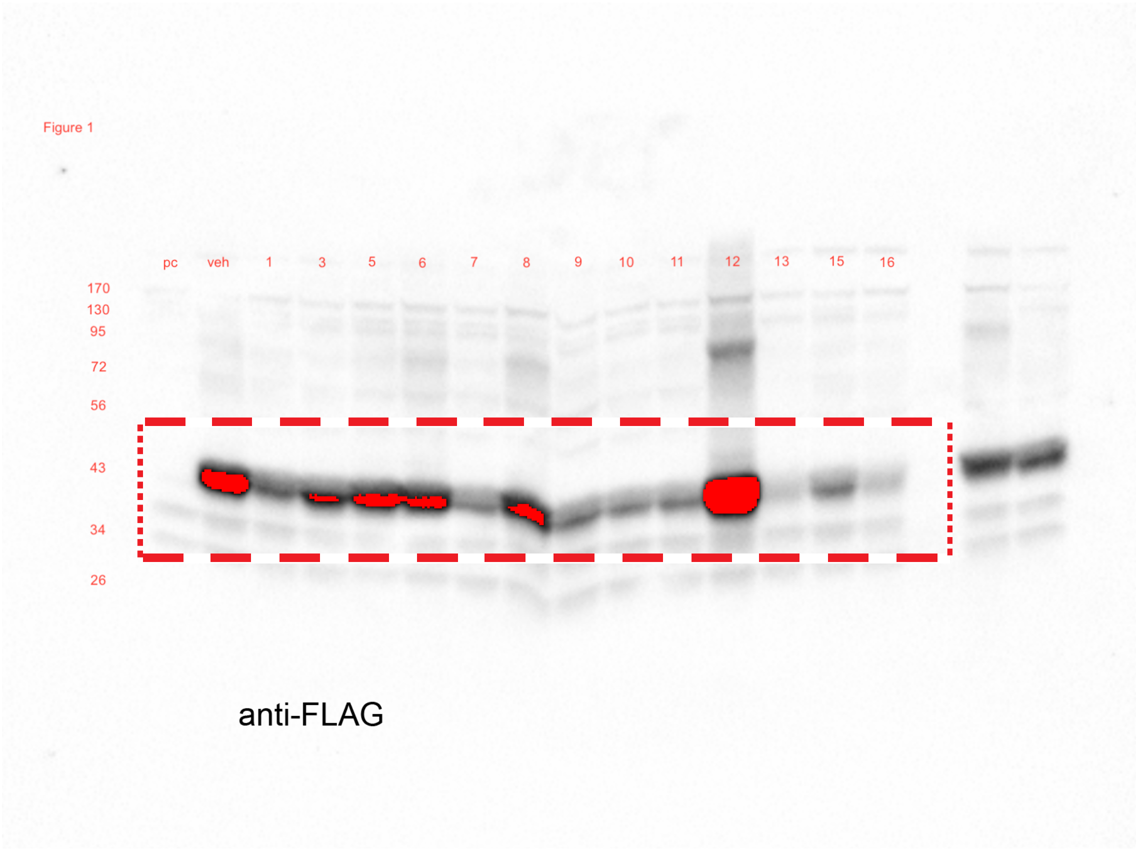
Figure 1
pc veh 1 3 5 6 7 8 9 10 11 12 13 15 16
170
130
95
72
56
43
34
26
anti-FLAG

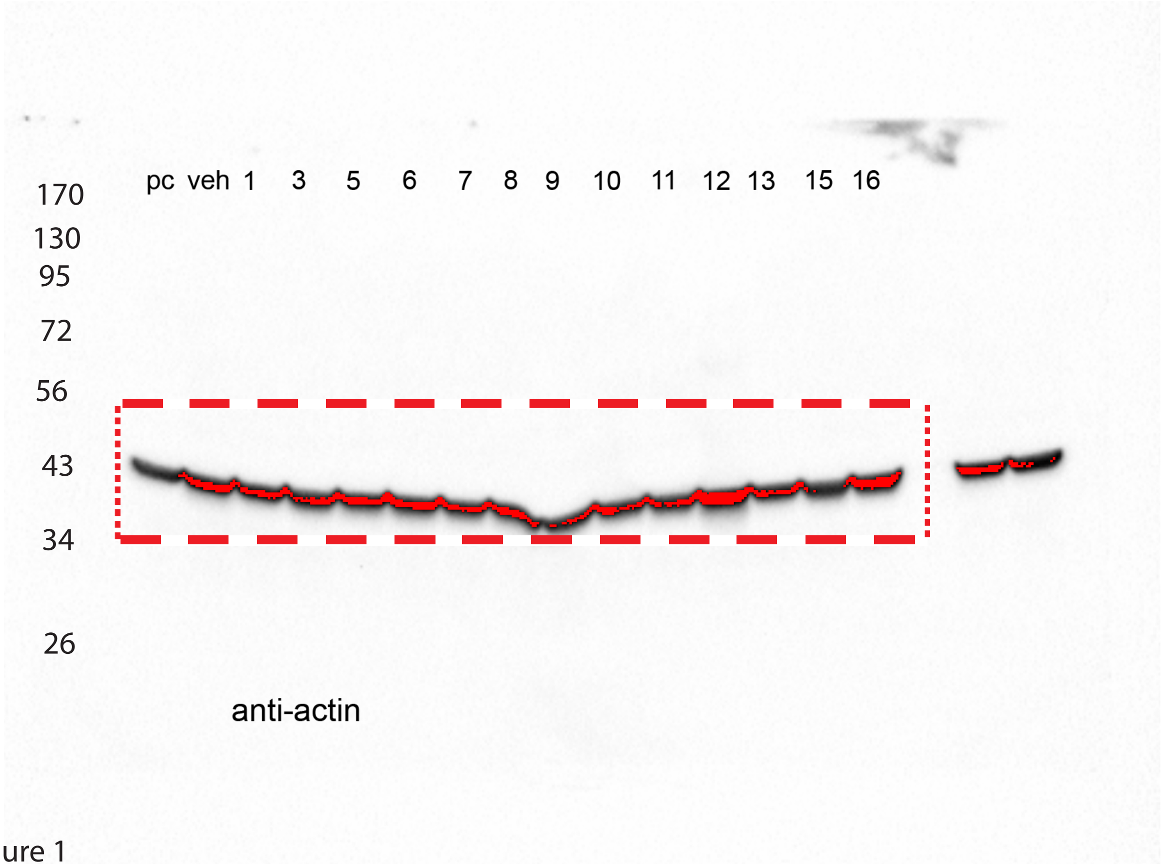
pc veh 1 3 5 6 7 8 9 10 11 12 13 15 16
170
130
95
72
56
43
34
26
anti-actin

Figure 1

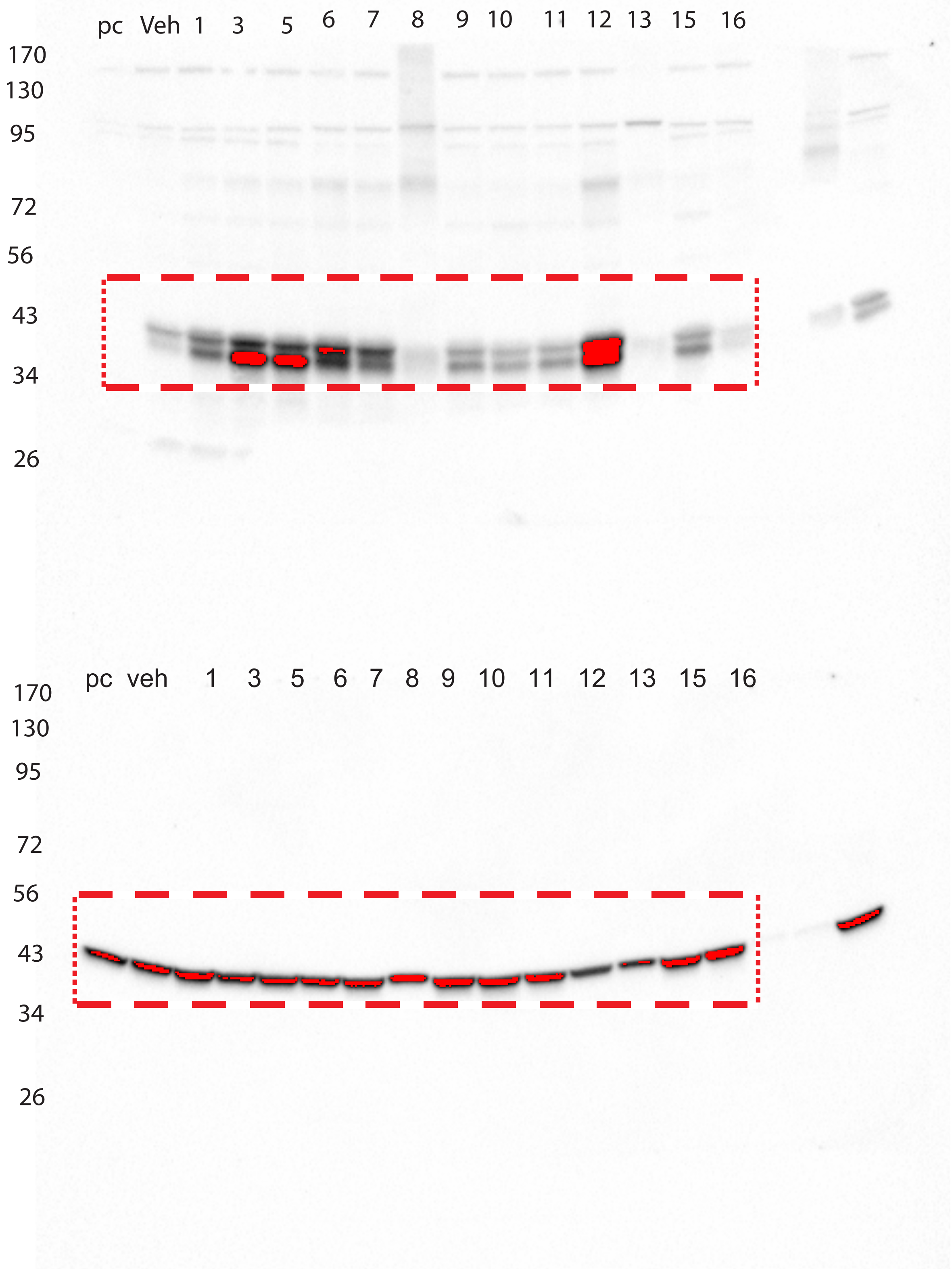
pc Veh 1 3 5 6 7 8 9 10 11 12 13 15 16
170
130
95
72
56
43
34
26
pc veh 1 3 5 6 7 8 9 10 11 12 13 15 16
170
130
95
72
56
43
34
26

Figure 1

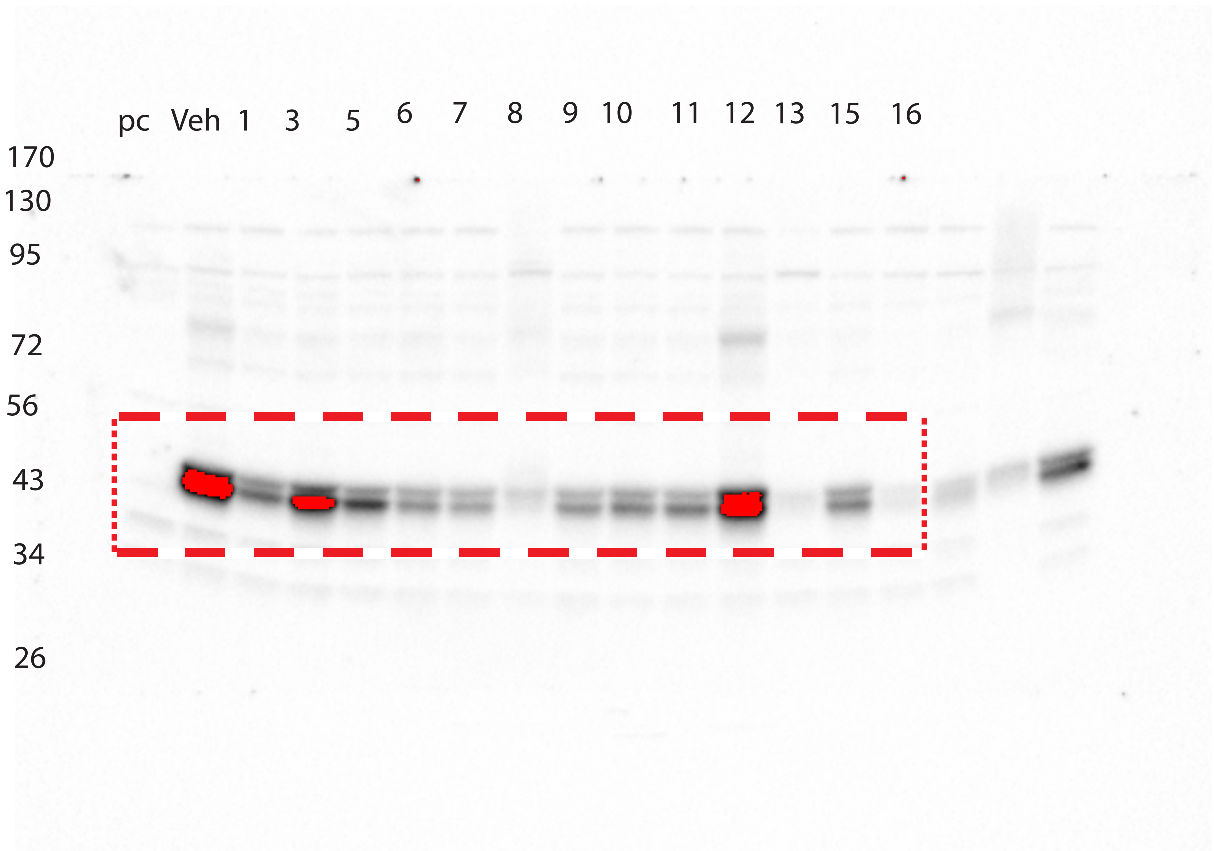
pc Veh 1 3 5 6 7 8 9 10 11 12 13 15 16
170
130
95
72
56
43
34
26

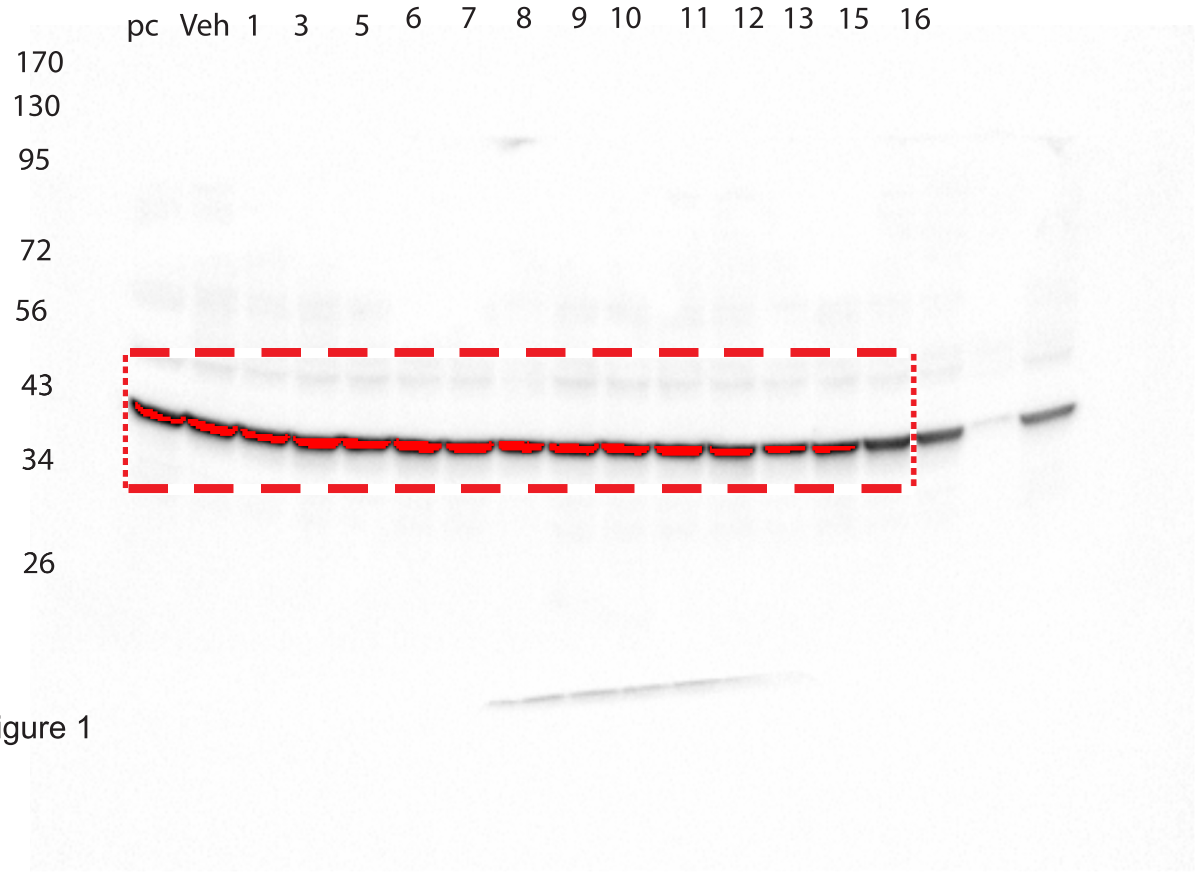
pc Veh 1 3 5 6 7 8 9 10 11 12 13 15 16
170
130
95
72
56
43
34
26

Figure 1

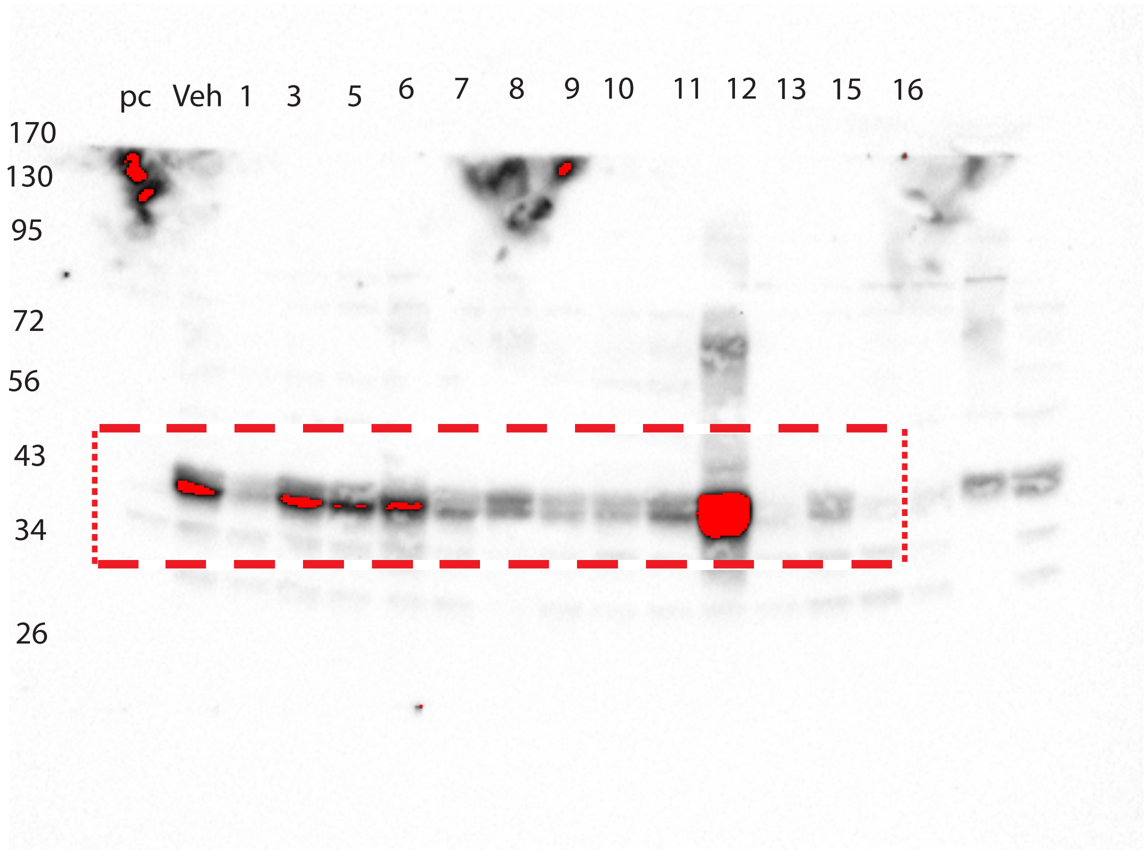
pc Veh 1 3 5 6 7 8 9 10 11 12 13 15 16
170
130
95
72
56
43
34
26

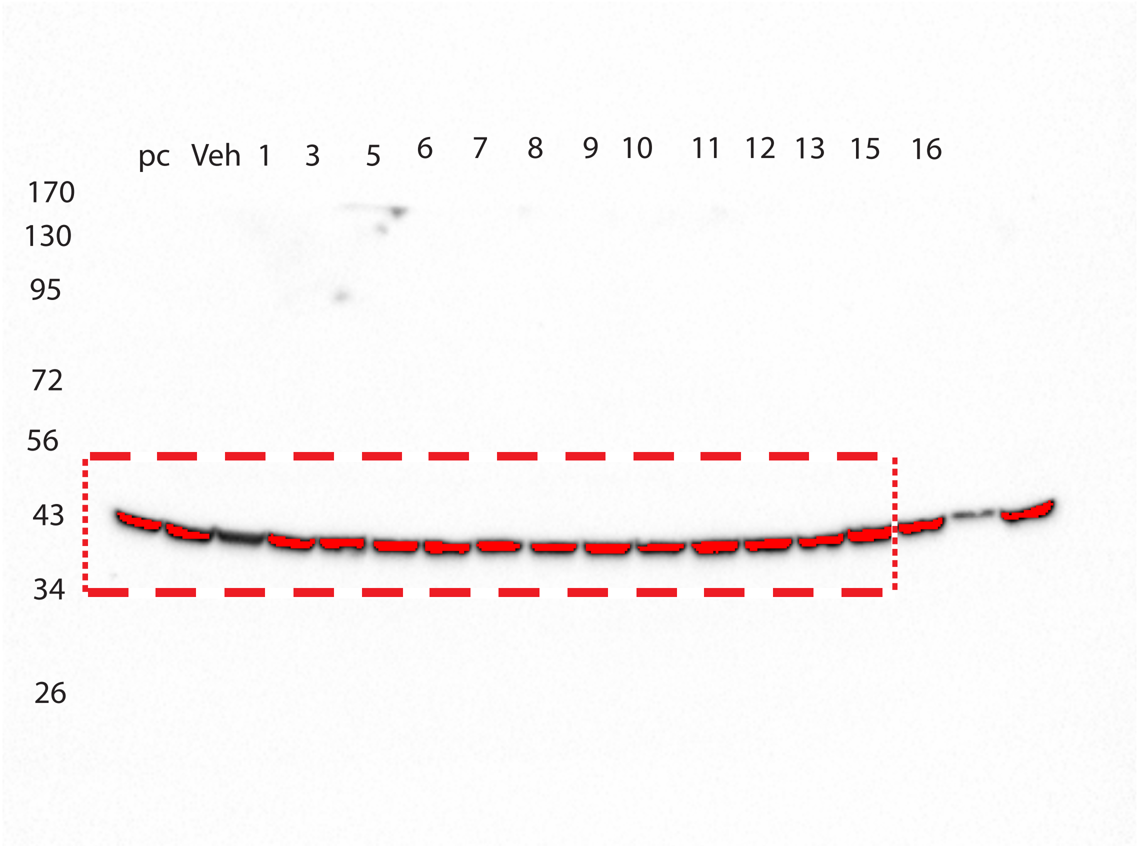
pc Veh 1 3 5 6 7 8 9 10 11 12 13 15 16
170
130
95
72
56
43
34
26

Figure 1

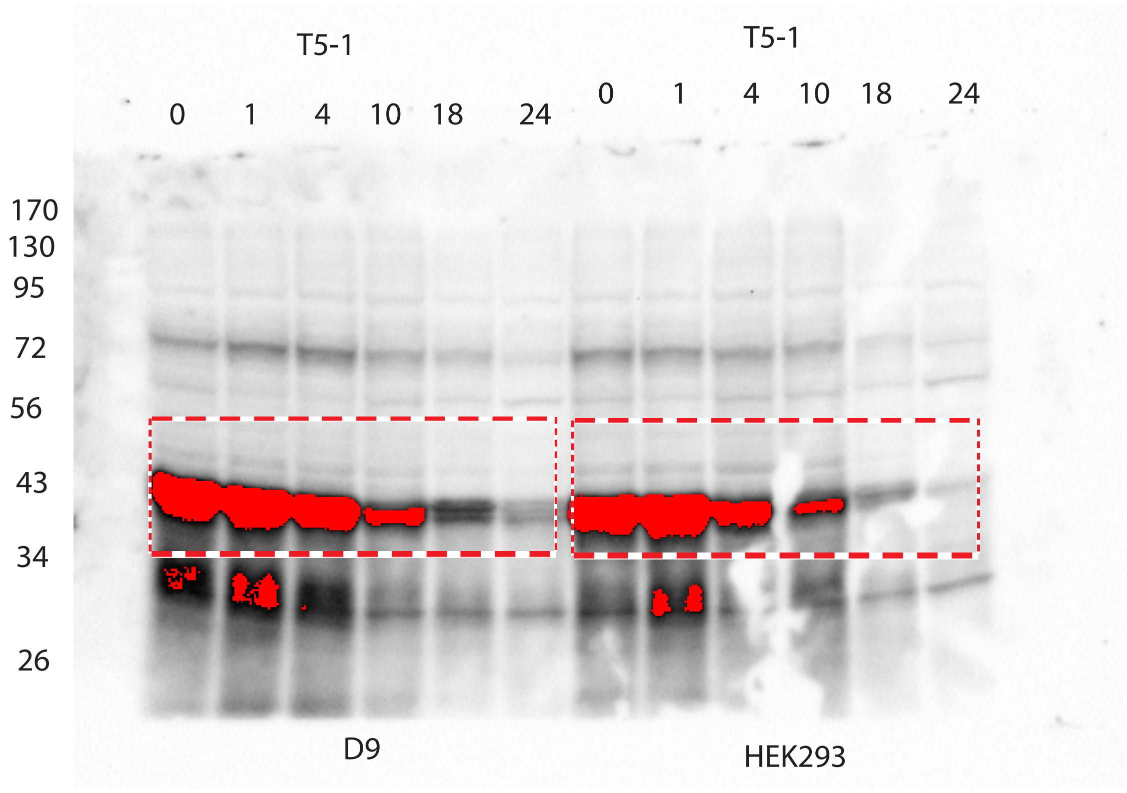
T5-1
0 1 4 10 18 24
T5-1
0 1 4 10 18 24
170
130
95
72
56
43
34
26
D9
HEK293

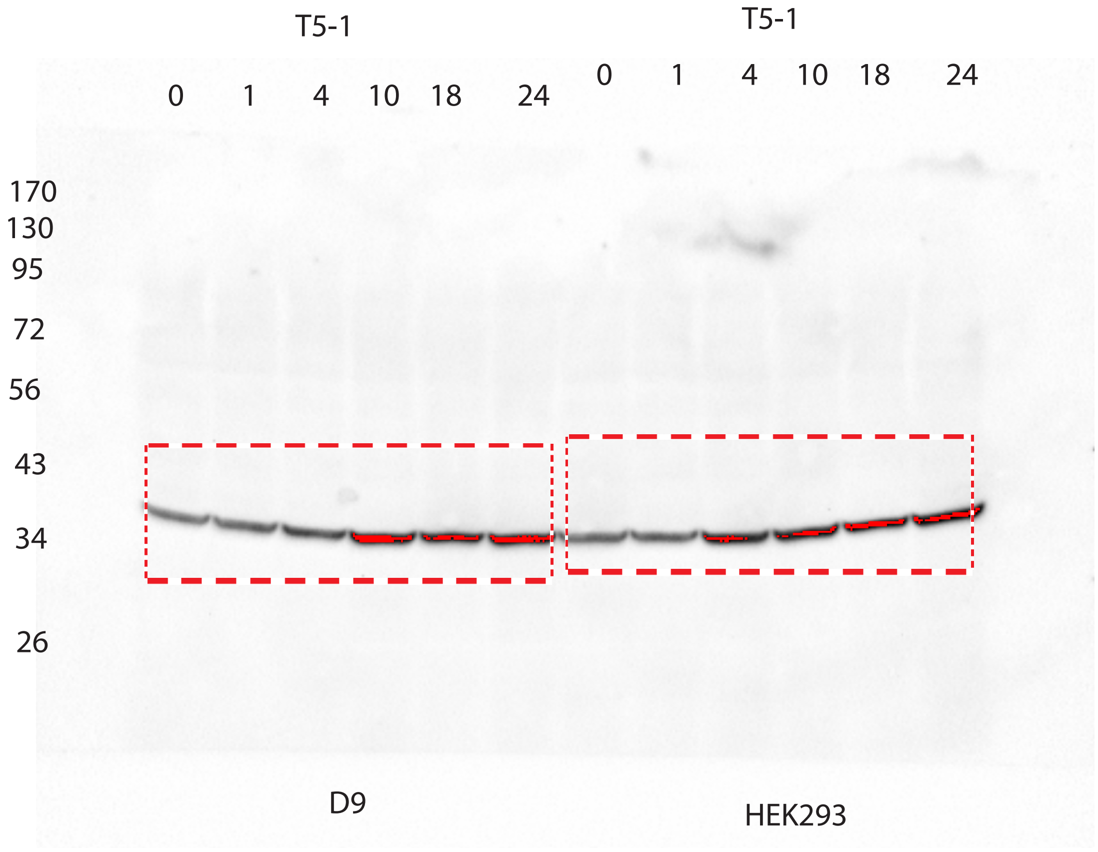
T5-1
0 1 4 10 18 24
T5-1
0 1 4 10 18 24
170
130
95
72
56
43
34
26
D9
HEK293

Fig 2 A and C

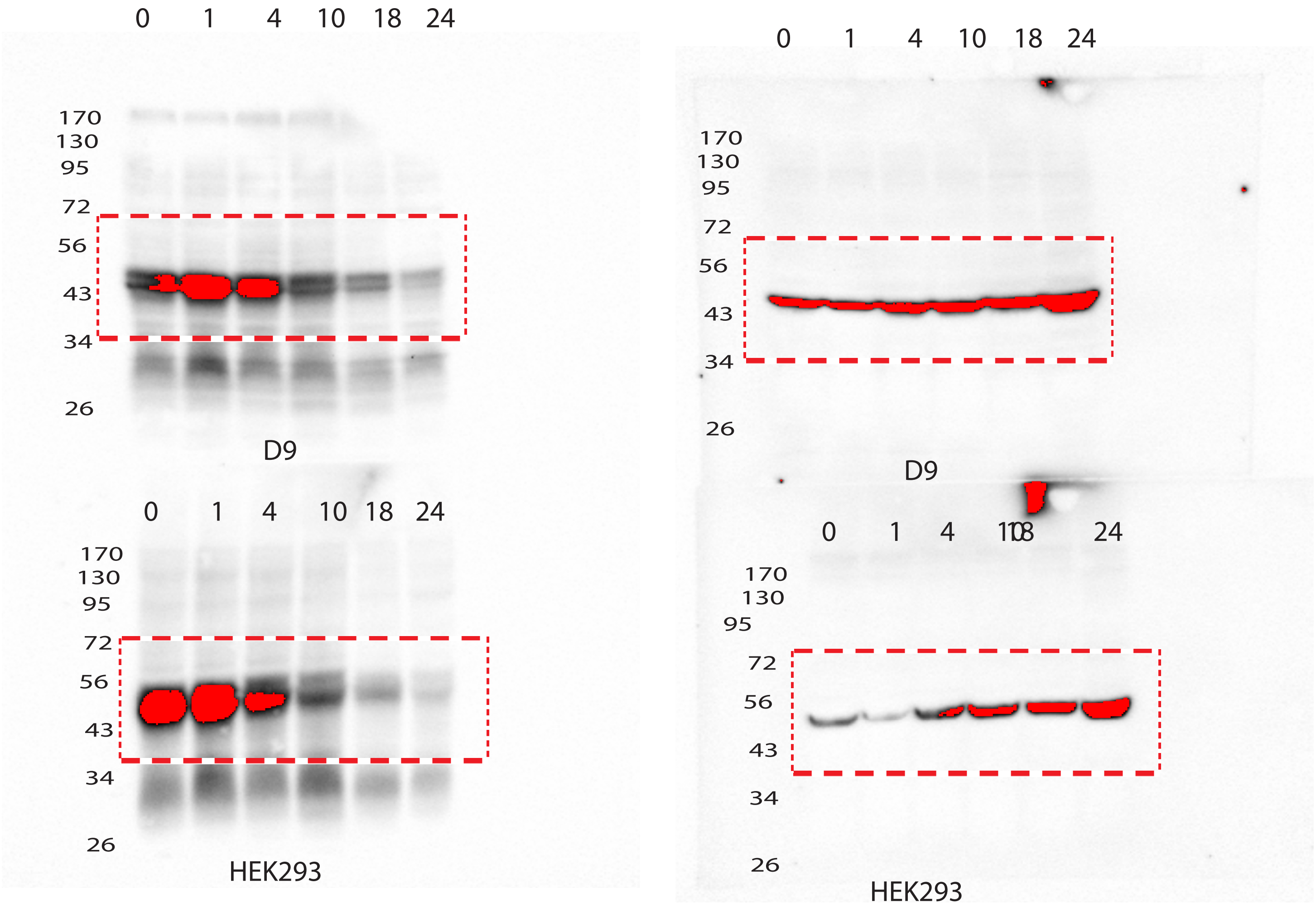

anti-FLAG

anti-actin

Fig 2 A and C

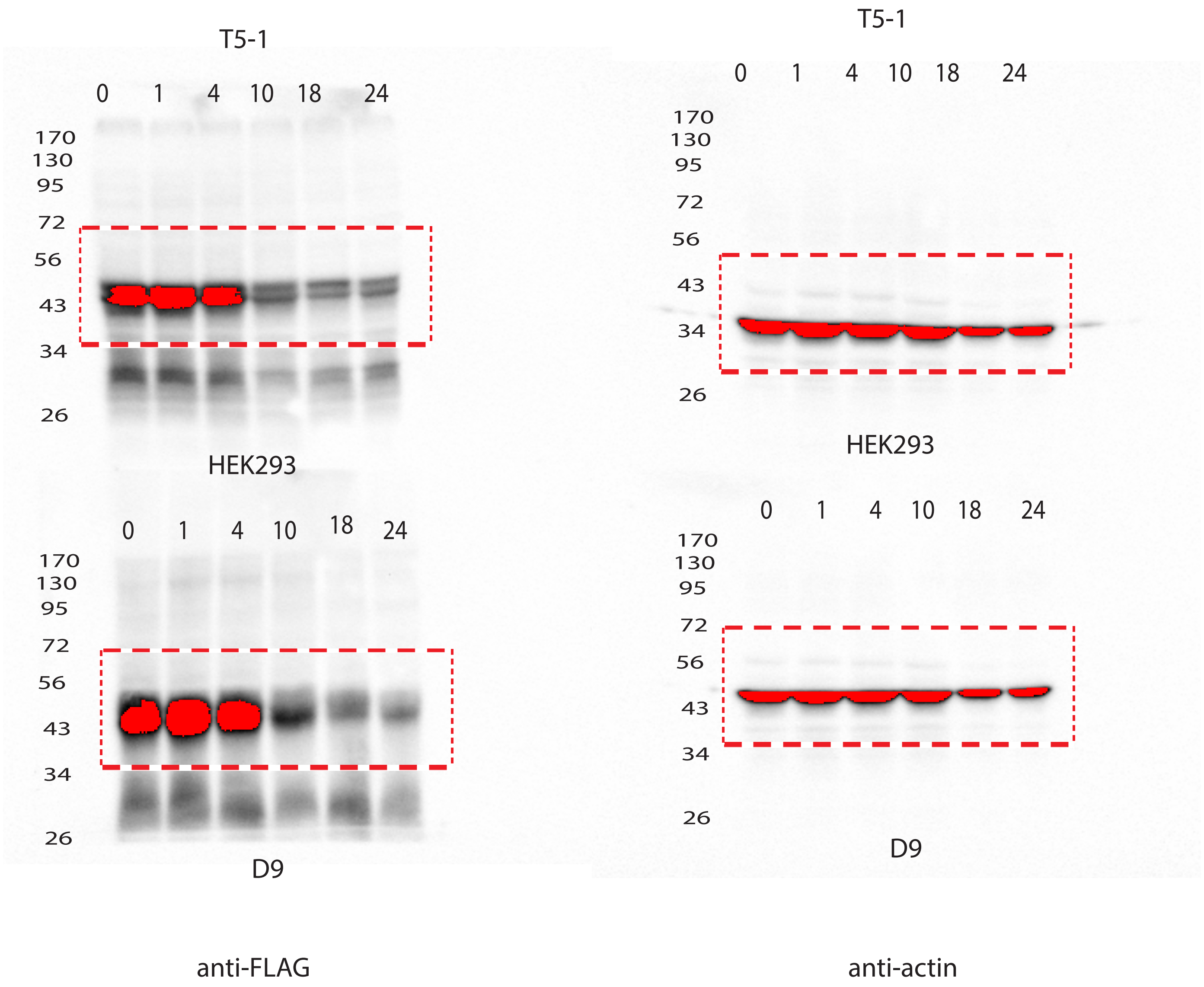
T5-1
0 1 4 10 18 24
170
130
95
72
56
43
34
26
HEK293
0 1 4 10 18 24
170
130
95
72
56
43
34
26
D9
T5-1
0 1 4 10 18 24
170
130
95
72
56
43
34
26
HEK293
0 1 4 10 18 24
170
130
95
72
56
43
34
26
D9
anti-FLAG
anti-actin

Fig 2 A and C

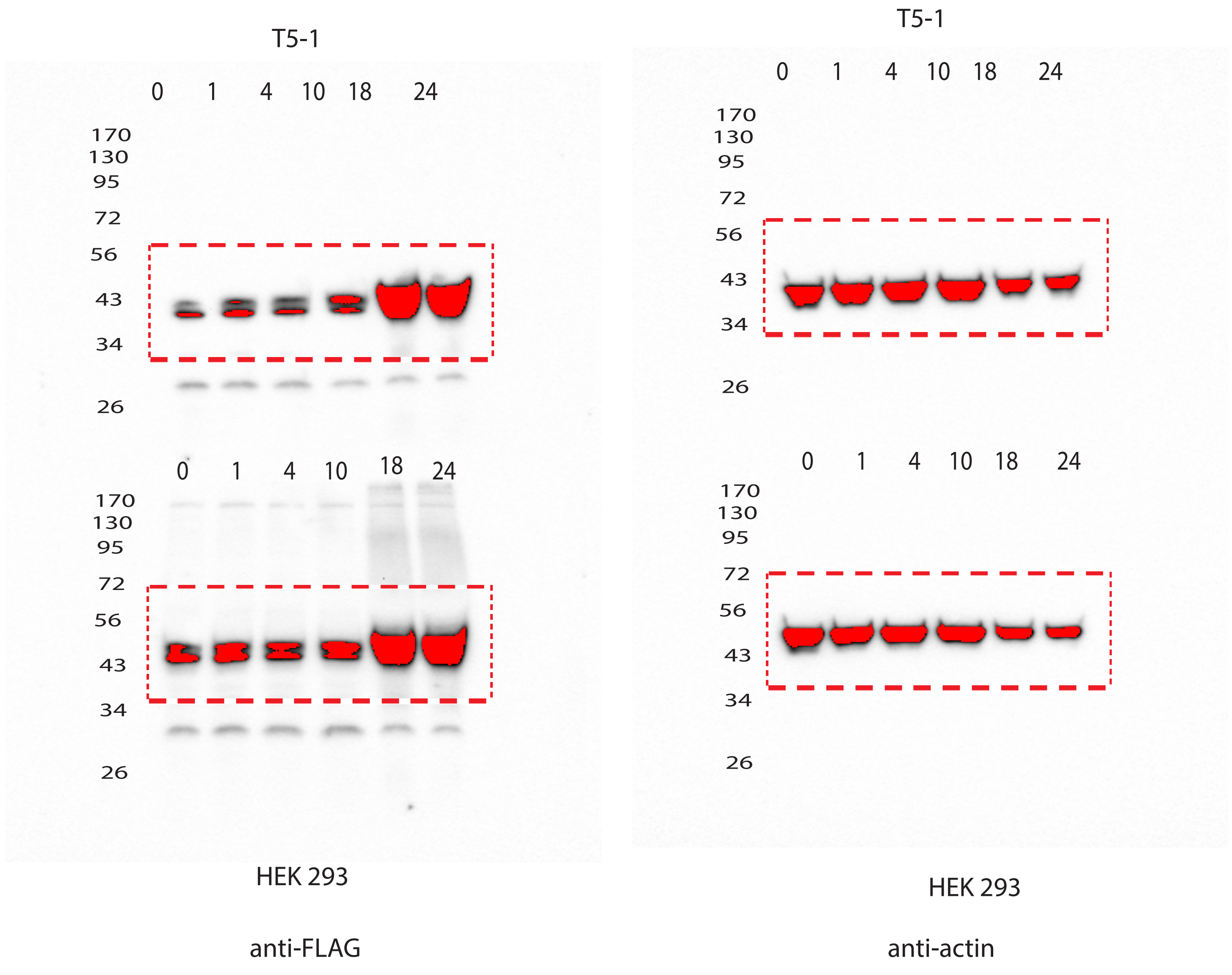
T5-1
0 1 4 10 18 24
170
130
95
72
56
43
34
26
0 1 4 10 18 24
170
130
95
72
56
43
34
26
HEK 293
anti-FLAG
T5-1
0 1 4 10 18 24
170
130
95
72
56
43
34
26
0 1 4 10 18 24
170
130
95
72
56
43
34
26
HEK 293
anti-actin

Fig 2 E and G

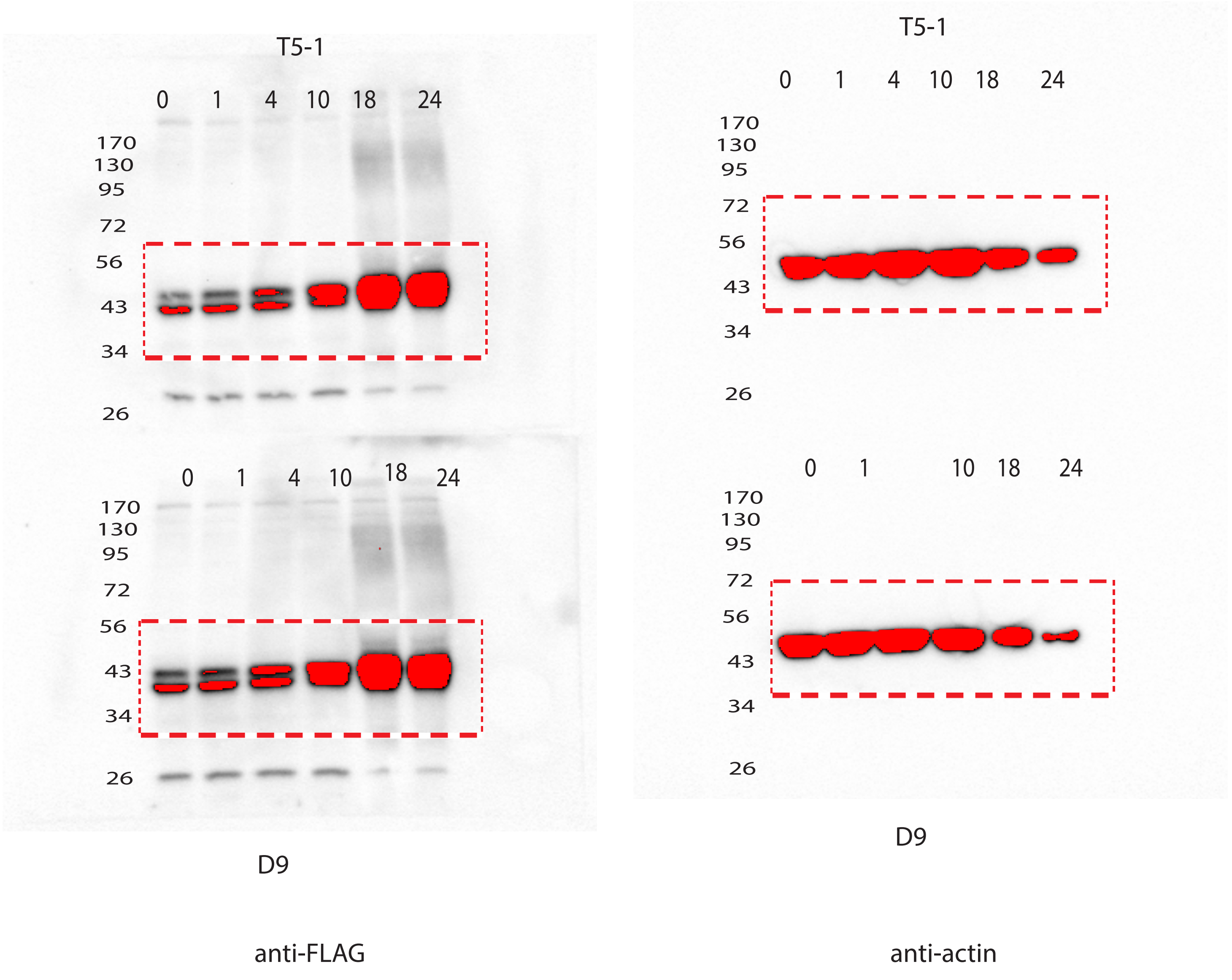
T5-1
0 1 4 10 18 24
170
130
95
72
56
43
34
26
0 1 4 10 18 24
170
130
95
72
56
43
34
26
D9
anti-FLAG
T5-1
0 1 4 10 18 24
170
130
95
72
56
43
34
26
0 1 10 18 24
170
130
95
72
56
43
34
26
D9
anti-actin

Fig 2 E and G

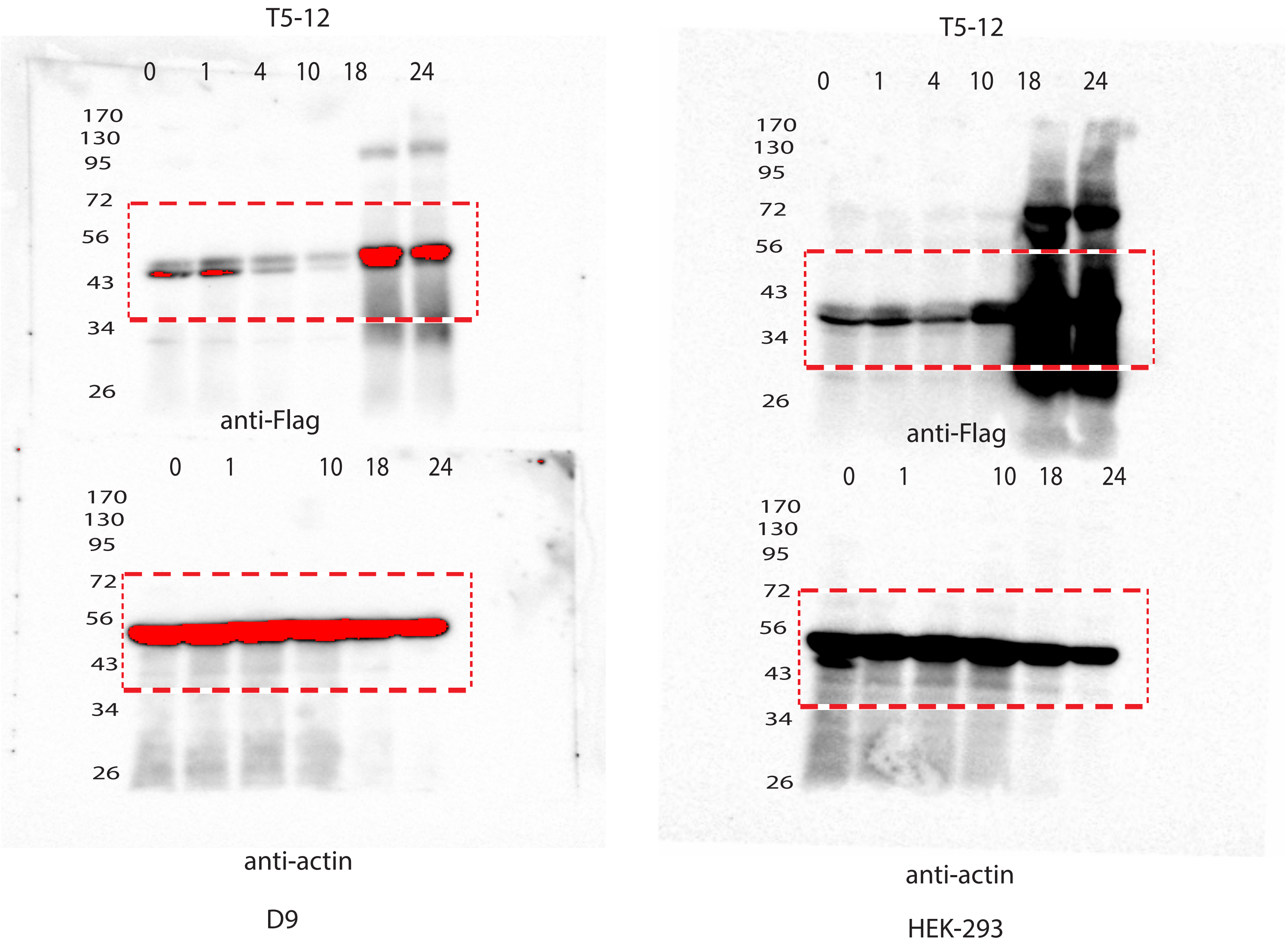
T5-12
0 1 4 10 18 24
170
130
95
72
56
43
34
26
anti-Flag
0 1 10 18 24
170
130
95
72
56
43
34
26
anti-actin
D9
T5-12
0 1 4 10 18 24
170
130
95
72
56
43
34
26
anti-Flag
0 1 10 18 24
170
130
95
72
56
43
34
26
anti-actin
HEK-293

Fig 2 E and G

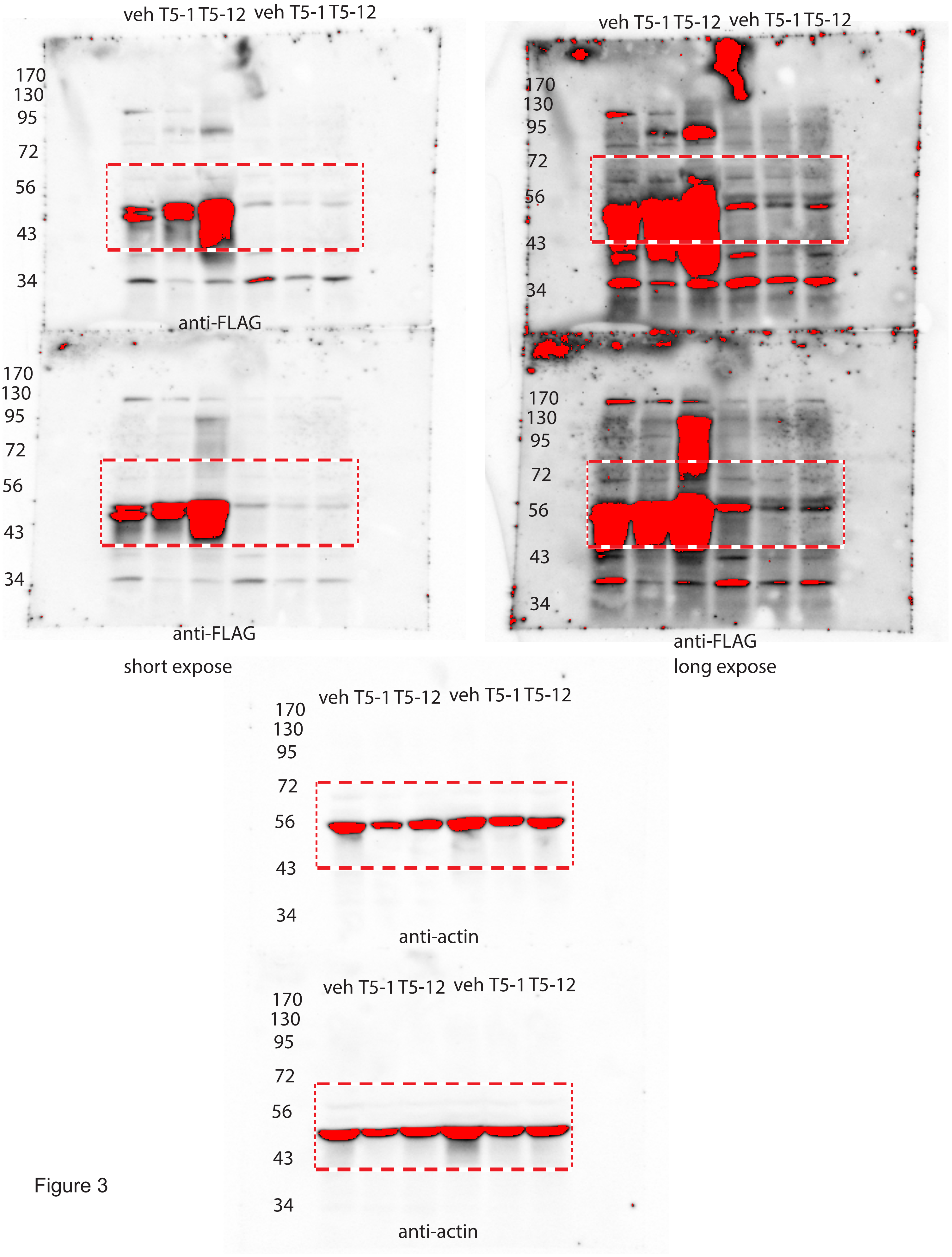
veh T5-1 T5-12 veh T5-1 T5-12
170
130
95
72
56
43
34
anti-FLAG
170
130
95
72
56
43
34
anti-FLAG
short expose
veh T5-1 T5-12 veh T5-1 T5-12
170
130
95
72
56
43
34
170
130
95
72
56
43
34
anti-FLAG
long expose
veh T5-1 T5-12 veh T5-1 T5-12
170
130
95
72
56
43
34
anti-actin
veh T5-1 T5-12 veh T5-1 T5-12
170
130
95
72
56
43
34
anti-actin

Figure 3

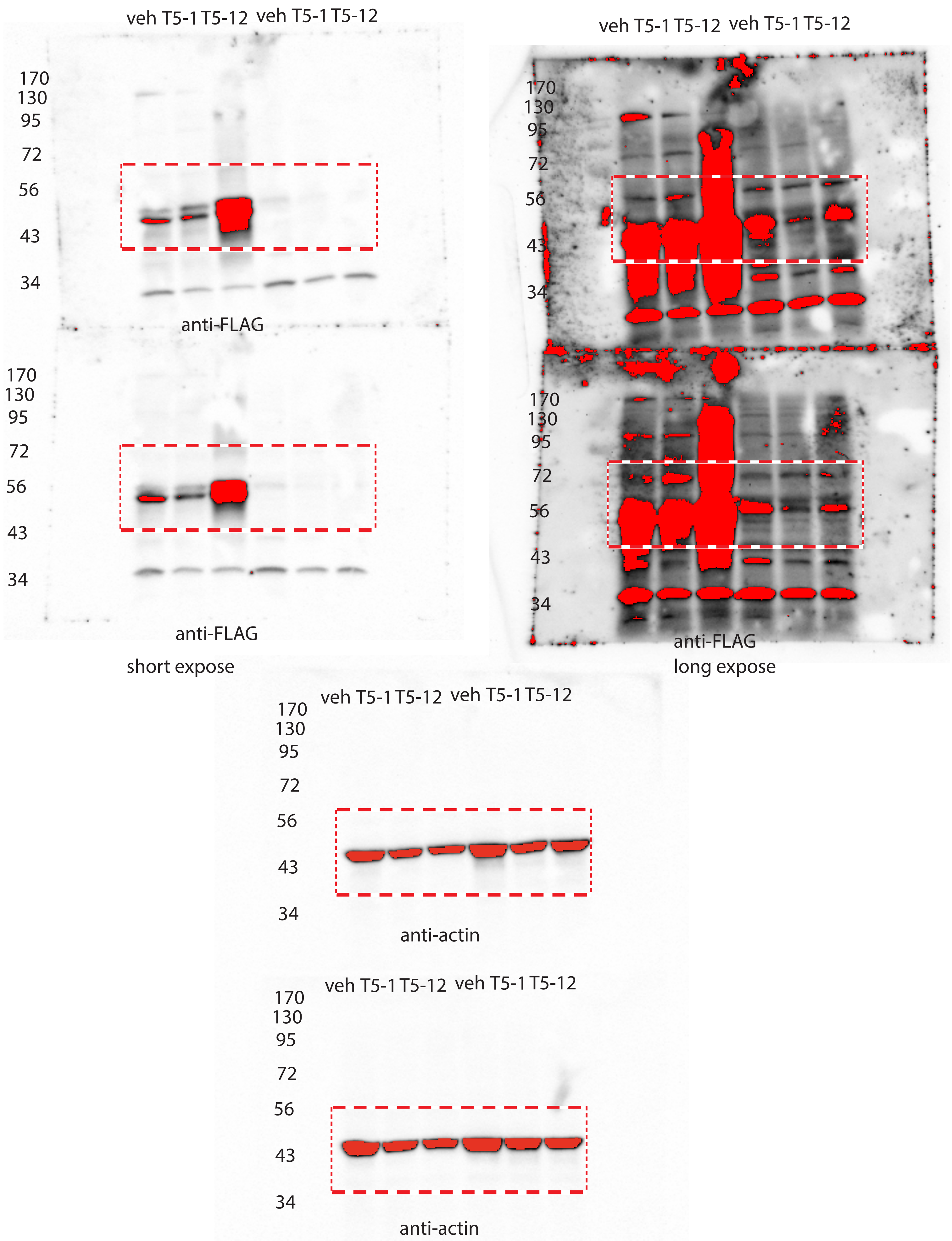
veh T5-1 T5-12 veh T5-1 T5-12
170
130
95
72
56
43
34
anti-FLAG
170
130
95
72
56
43
34
anti-FLAG
short expose
veh T5-1 T5-12 veh T5-1 T5-12
170
130
95
72
56
43
34
170
130
95
72
56
43
34
anti-FLAG
long expose
veh T5-1 T5-12 veh T5-1 T5-12
170
130
95
72
56
43
34
anti-actin
veh T5-1 T5-12 veh T5-1 T5-12
170
130
95
72
56
43
34
anti-actin

Figure 3

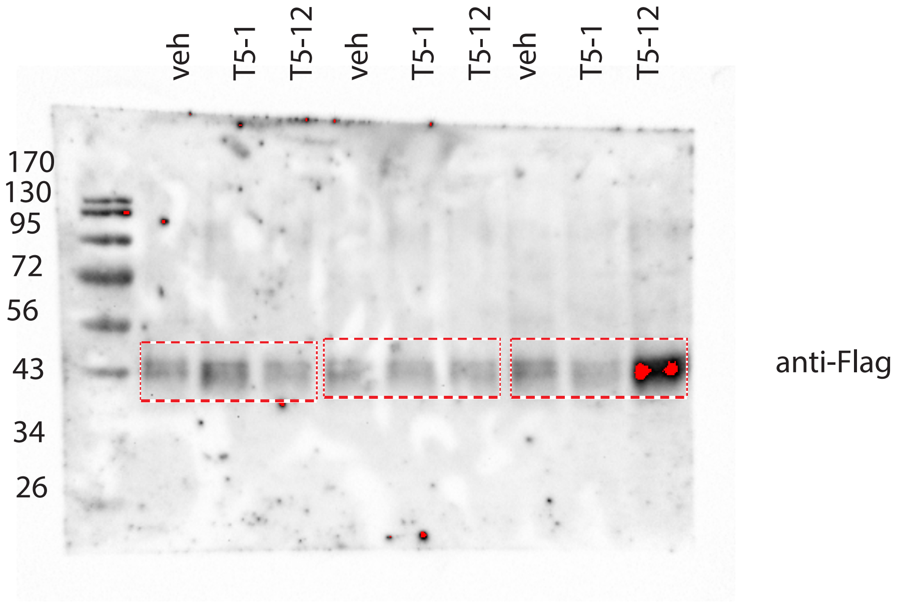
veh
T5-1
T5-12
veh
T5-1
T5-12
veh
T5-1
T5-12
170
130
95
72
56
43
34
26
anti-Flag

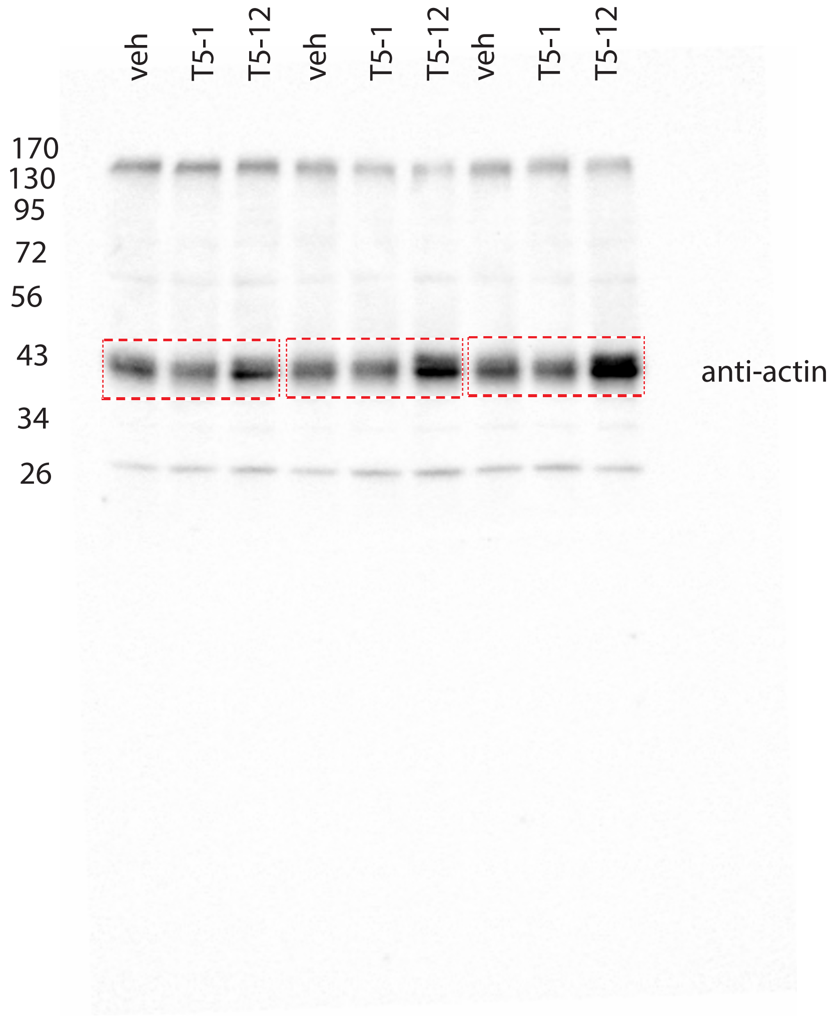
veh
T5-1
T5-12
veh
T5-1
T5-12
veh
T5-1
T5-12
170
130
95
72
56
43
34
26
anti-actin

Figure 4

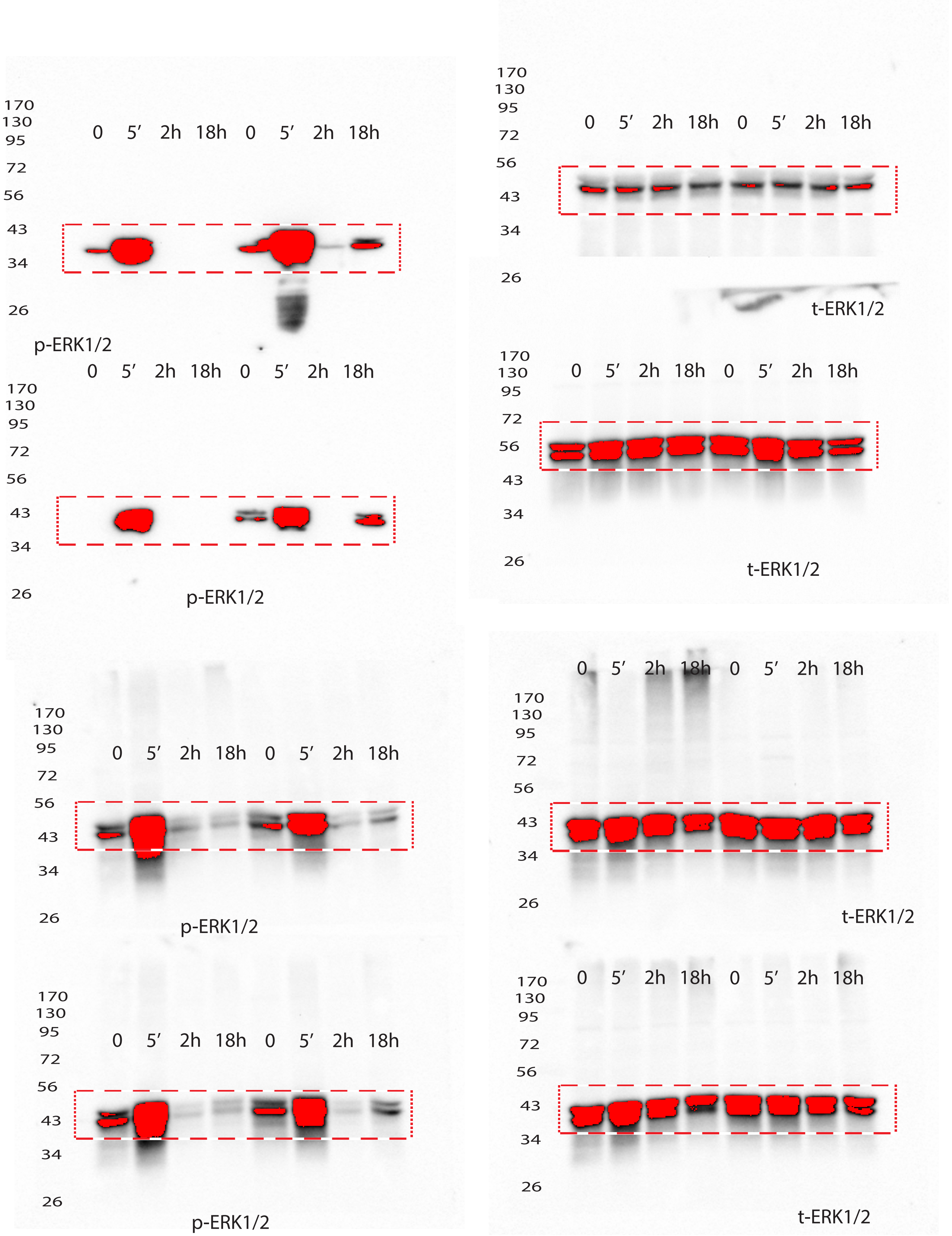
170
130
95
72
56
43
34
26
0 5′ 2h 18h 0 5′ 2h 18h
p-ERK1/2
170
130
95
72
56
43
34
26
0 5′ 2h 18h 0 5′ 2h 18h
t-ERK1/2
0 5′ 2h 18h 0 5′ 2h 18h
170
130
95
72
56
43
34
26
p-ERK1/2
170
130
95
72
56
43
34
26
0 5′ 2h 18h 0 5′ 2h 18h
t-ERK1/2
170
130
95
72
56
43
34
26
0 5′ 2h 18h 0 5′ 2h 18h
p-ERK1/2
0 5′ 2h 18h 0 5′ 2h 18h
170
130
95
72
56
43
34
26
t-ERK1/2
170
130
95
72
56
43
34
26
0 5′ 2h 18h 0 5′ 2h 18h
p-ERK1/2
170
130
95
72
56
43
34
26
0 5′ 2h 18h 0 5′ 2h 18h
t-ERK1/2

Figure 5

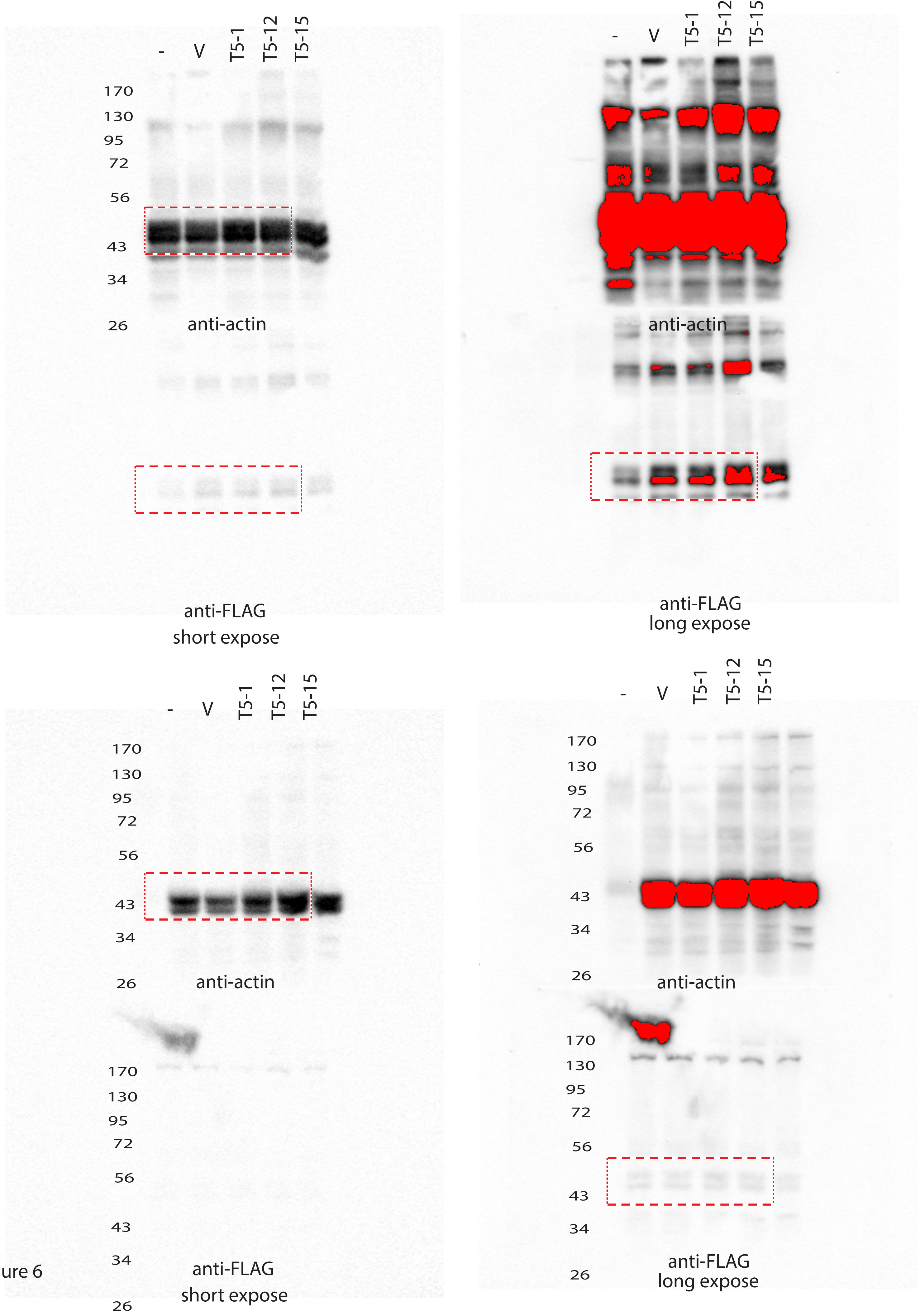

-
V
T5-1
T5-12
T5-15
170
130
95
72
56
43
34
26
anti-actin
anti-FLAG
short expose
-
V
T5-1
T5-12
T5-15
anti-actin
anti-FLAG
long expose
-
V
T5-1
T5-12
T5-15
170
130
95
72
56
43
34
26
anti-actin
170
130
95
72
56
43
34
26
anti-FLAG
short expose
-
V
T5-1
T5-12
T5-15
170
130
95
72
56
43
34
26
anti-actin
170
130
95
72
56
43
34
26
anti-FLAG
long expose

Figure 6

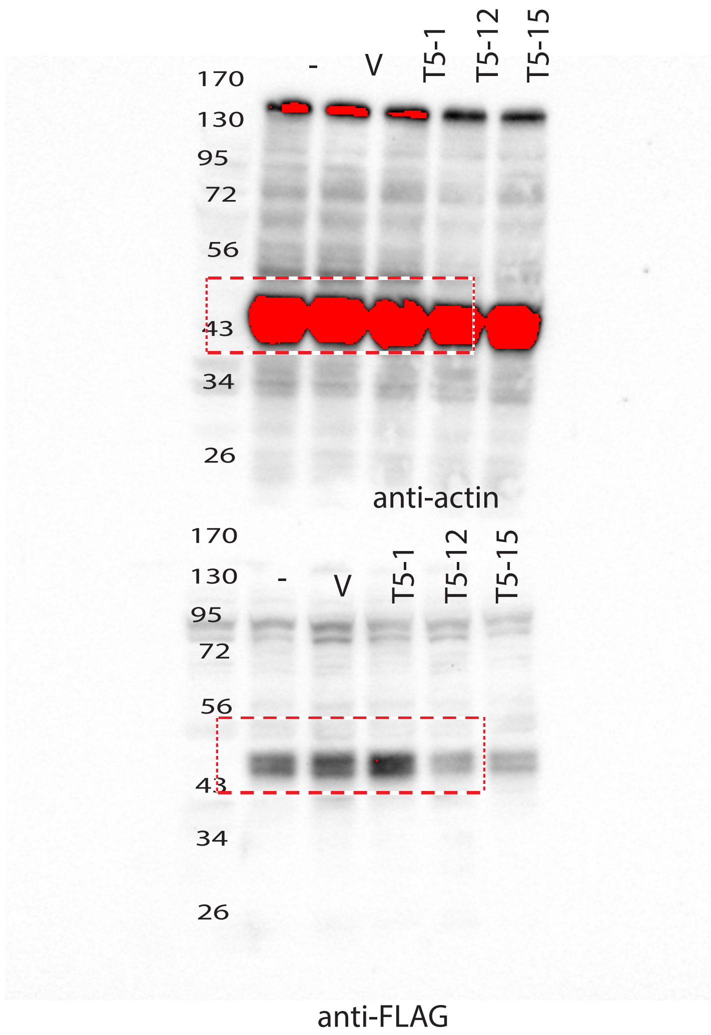
- V T5-1 T5-12 T5-15
170
130
95
72
56
43
34
26
anti-actin
170
130
95
72
56
43
34
26
- V T5-1 T5-12 T5-15
anti-FLAG

Figure 6
